# SQLE Knockdown inhibits bladder cancer progression by regulating the PTEN/AKT/GSK3β signaling pathway through P53

**DOI:** 10.1186/s12935-023-02997-5

**Published:** 2023-09-28

**Authors:** Fan Zou, Wu Chen, Tianbao Song, Ji Xing, Yunlong Zhang, Kang Chen, Weimin Hu, Linzhi Li, Jinzhuo Ning, Chenglong Li, Weimin Yu, Fan Cheng

**Affiliations:** 1 Department of Urology, Renmin Hospital of Wuhan University, 99 ziyang road, Wuhan, 430060 Hubei Province China; 2 Department of Urology, Tongji Medical College, Union Hospital, Huazhong University of Science and Technology, Wuhan, 430022 China

**Keywords:** Bladder cancer, SQLE, P53, PTEN/AKT/GSK3β signaling pathway, Apoptosis, Cell cycle, Proliferation

## Abstract

Bladder cancer (BCa) is one of the most common malignancies worldwide. However, the lack of accurate and effective targeted drugs has become a major problem in current clinical treatment of BCa. Studies have demonstrated that squalene epoxidase (SQLE), as a key rate-limiting enzyme in cholesterol biosynthesis, is involved in cancer development. In this study, our analysis of The Cancer Genome Atlas, The Genotype-Tissue Expression, and Gene Expression Omnibus databases showed that SQLE expression was significantly higher in cancer tissues than it was in adjacent normal tissues, and BCa tissues with a high SQLE expression displayed a poor prognosis. We then confirmed this result in qRT-PCR and immunohistochemical staining experiments, and our vitro studies demonstrated that SQLE knockdown inhibited tumor cell proliferation and metastasis through the PTEN/AKT/GSK3β signaling pathway. By means of rescue experiments, we proved that that P53 is a key molecule in SQLE-mediated regulation of the PTEN/AKT/GSK3β signaling pathway. Simultaneously, we verified the above findings through a tumorigenesis experiment in nude mice. In conclusion, our study shows that SQLE promotes BCa growth through the P53/PTEN/AKT/GSK3β axis, which may serve as a therapeutic biological target for BCa.

**Supplementary Information:**

The online version contains supplementary material available at 10.1186/s12935-023-02997-5.

## Introduction

Bladder cancer (BCa) is one of the most prevalent malignant tumors worldwide, with an estimated 573,278 new cases and 199,922 fatalities all over the world in 2020 [1]. Compared with breast cancer [2], lung cancer [3, 4], and prostate cancer [5], the treatment of BCa is relatively limited. Especially for advanced BCa, we lack effective treatments to postpone the further spread of tumor cells. Therefore, it is necessary for us to identify new molecular targets that can effectively reduce the growth rate of tumor cells and prevent non-muscle-invasive bladder cancer (NMIBC) from progressing to muscle-invasive bladder cancer (MIBC).

For a long time, obesity and cholesterol metabolism disorder have been regarded as potential risk factors for cancer [6]. Meanwhile, aberrant lipid metabolism is increasingly considered a target characteristic of different cancer types. Studies have confirmed that lipid accumulation is primarily regulated by SREBF family transcription factors [7], which aberrantly activate a bunch of signal pathways, such as the phosphatidylinositol-3 kinase (PI3K)/AKT signaling pathway [8–10] and the MAP kinase (MAPK) signaling pathway [11], which play a vital role in promoting tumor cell growth, migration, and invasion. However, how abnormal lipid metabolism affects these processes remains to be ascertained.

Squalene epoxidase (SQLE), also known as squalene monooxygenase, is a key rate-limiting enzyme in cholesterol biosynthesis [12]. It plays a key role in catalyzing stereospecific conversion of non-sterol intermediate squalene to 2,3(*S*)-oxidosqualene [13]. As an important enzyme of lipid metabolism, SQLE is a protein composed of 574 amino acids that is located at chromosome 8q24.13 and spans approximately 23.8 kilobase pairs [14]. SQLE affects the growth and development of normal cells as well as tumor cells. Nelson et al. [15] reported that 27-hydroxycholesterol (27HC), a cholesterol metabolite, can promote tumor growth and development by estrogen receptor (ER) and the liver X receptor (LXR) modulation. Their finding highlights the potential role of SQLE in the growth of tumor cells.

Although SQLE has been shown as an oncogene that is overexpressed in several cancers, including hepatocellular carcinoma [16] (HCC) and colorectal cancer [17] (COAD), and overexpression of SQLE tends to bring a bad prognosis in these cancers, the interaction between BCa and SQLE remains poorly understood. In this study, Using The Cancer Genome Atlas (TCGA) and Gene Expression Omnibus (GEO), we found that SQLE was upregulated in BCa compared with adjacent normal tissues, and the elevated expression level of SQLE predicted a poor prognosis. To comprehensively understand the role of SQLE in BCa, we established stably transfected cell lines in vitro, with which the growth and infiltration rate of BCa cells were activated remarkably. After that, we uncovered a novel mechanism that SQLE could promote the growth and metastasis of BCa cell via the P53/PTEN/AKT signaling pathway, a vital pathway regulating glycolysis, cell cycle, apoptosis, and various other biological functions. Herein, we demonstrate this in vivo through tumor formation experiments in nude mice. Our results provide a basis for investigating SQLE in current and future designs of targeted therapy against BCa.

## Materials and methods

### Data acquisition and differential expression analysis

The Cancer Genome Atlas (TCGA, http://cancergenome.nih.gov) and Genotype-Tissue Expression (GTEx, https://gtexportal.org/) datasets were obtained from UCSC Xena browser (https://xenabrowser.net/), with 28 adjacent normal tissues and 404 tumor tissues from RNA-sequencing and clinical data. GSE13507 and GSE188715 consist of 256 tissues, and 70 tissues were accessed from the Gene Expression Omnibus (GEO) dataset. Subsequently, the RNA sequencing data were normalized to log2 (TPM + 1). The R software (version 4.2.1) was used to perform statistical analyses. The “Wilcox.test” algorithm was used to analyze the differences of SQLE expression between tumor samples and adjacent normal tissues in BCa, and differences with p < 0.05 were considered statistically significant. Patient samples with histologically confirmed BCa were collected in People’s hospital of Wuhan University when patients underwent surgery (n = 9). Patient information is encapsulated in Table 1. Both tumor and adjacent normal tissues were immediately snap-frozen in liquid nitrogen. This study has been approved by the Ethics Committee of Medical School of Wuhan University.

**Table 1:** Clinicopathological characteristics of nine patients with BCa.

Variable	Groups	Total
Gender	Male	7
	Female	2
Age (years)	≥ 65	6
	< 65	3
Multiplicity of tumor	Single	2
	Multiple	7
Grade	Low grade	1
	High grade	8
Stage	Ta, T1	1
	T2–T4	8
Lymph nodes	Negative	4
	Positive	5
Distant metastasis	Absent	0
	Present	9

### Diagnostic and clinical prognostic analysis of SQLE

Using the “SurvMiner” and “Survival” packages, the median expression values based on SQLE were classified into two groups, namely, the high-risk and low-risk groups in BCa. The R software was also used to draw Kaplan-Meier curves, and their statistical significance was calculated using the log-rank test.

### Genetic alteration and immune infiltration analysis of SQLE

In this study, we searched the cBioPortal database [18] (https://www.cbioportal.org/) for genetic alteration information of SQLE through the “Mutations” and “Plots” modules. “ggExtra” and “ggpubr” packages were used to draw scatter diagrams. “Estimate” package was utilized to ascertain the correlation of SQLE expression with immune score, stromal score, and ESTIMATE score. From the TIMER2.0 database (http://timer.compgenomics.org/), we downloaded the infiltration scores of various immune cells, including B cells, CD4 + T cells, CD8 + T cells, macrophages, and myeloid-derived suppressor cells.

### The construction of protein–protein Interaction network and functional enrichment analysis

The data of potential protein–protein interactions (PPIs) with SQLE were calculated using the “Pearson” algorithm. The top 170 genes were imported into the STRING database (https://string-db.org/) and Cytoscape (v3.8.2) for subsequent analysis and visualization. Meanwhile, the PPI network were uploaded to the Database for Annotation, Visualization, and Integrated Discovery (DAVID, https://david.ncifcrf.gov/home.jsp). Finally, Gene Ontology (GO) analysis and Kyoto Encyclopedia of Genes and Genomes (KEGG) pathway analysis enrichment results were obtained.

### Drug and antibodies

PFT-β hydrobromide (purity of 99.97%, PFT-β), an inhibitor of the p53 protein [19], was purchased from MedChemExpress (Shanghai, China) and dissolved in dimethyl sulfoxide (DMSO) to a certain concentration. The following antibodies were used in the present study: SQLE (TD12063, Abmart), KI67 (27309-1-AP, Prointech), P-AKT (66444-1-Ig, Proteintech), AKT (60203-2-Ig, Proteintech), P-GSK-3β (#9323, CST), GSK-3β (#9832, CST), PTEN (60300-1-Ig, Proteintech), P53 (ab26, Abcam), Cyclin D1 (26939-1-AP, Proteintech), Bcl-2 (#15,071, CST), Bax (60267-1-Ig, Proteintech), cleaved-Caspase-3 (#9664, CST), and GAPDH (60004-1-Ig, Proteintech). More detailed antibody information is presented in Table 2.

**Table 2:** Details of antibodies

Antibodies	Company	Catalog number	Source	Dilutions
SQLE	Abmart	TD12063	Rabbit	WB:1:1000 IF/IHC:1:100
KI67	Proteintech	27309-1-AP	Rabbit	1:200
Phospho-AKT	Proteintech	66444-1-Ig	Mouse	WB:1:10000 IF/IHC:1:100
AKT	Proteintech	60203-2-Ig	Mouse	1:10000
Phospho-GSK-3β	Cell Signalling Technology	#9323	Rabbit	1:1000
GSK-3β	Cell Signalling Technology	#9832	Mouse	1:1000
PTEN	Proteintech	60300-1-Ig	Mouse	WB:1:1000 IF:1:200
P53	Abcam	ab26	Mouse	WB/IF:1 µg/ml
cyclin D1	Proteintech	26939-1-AP	Rabbit	WB:1:10000 IHC:1:500
Bcl-2	Cell Signalling Technology	#15,071	Mouse	1:1000
Bax	Proteintech	60267-1-Ig	Mouse	WB:1:5000 IHC:1:500
Cleaved-Caspase-3	Cell Signalling Technology	#9664	Rabbit	1:1000
GAPDH	Proteintech	60004-1-Ig	Mouse	1:20000

### Cell culture

BCa cell lines (T24 and 5637) and normal ureteral epithelial cells (SV-HUC-1) were acquired from the Cell Bank of the Chinese Academy of Sciences (Shanghai, China). All BCa cells were cultured in the RPMI-1640 medium (HyClone, Logan, UT, USA) with 1% sodium penicillin G/streptomycin sulfate and 10% fetal bovine serum (FBS; Gibco, Waltham, MA, USA), and SV-HUC-1 cells were incubated in the F12K medium (HyClone, Logan, UT, USA) containing 10% FBS. A humidified chamber containing 5% CO_2_ was used to incubate all cells at 37 °C.

**Fig. 1:**
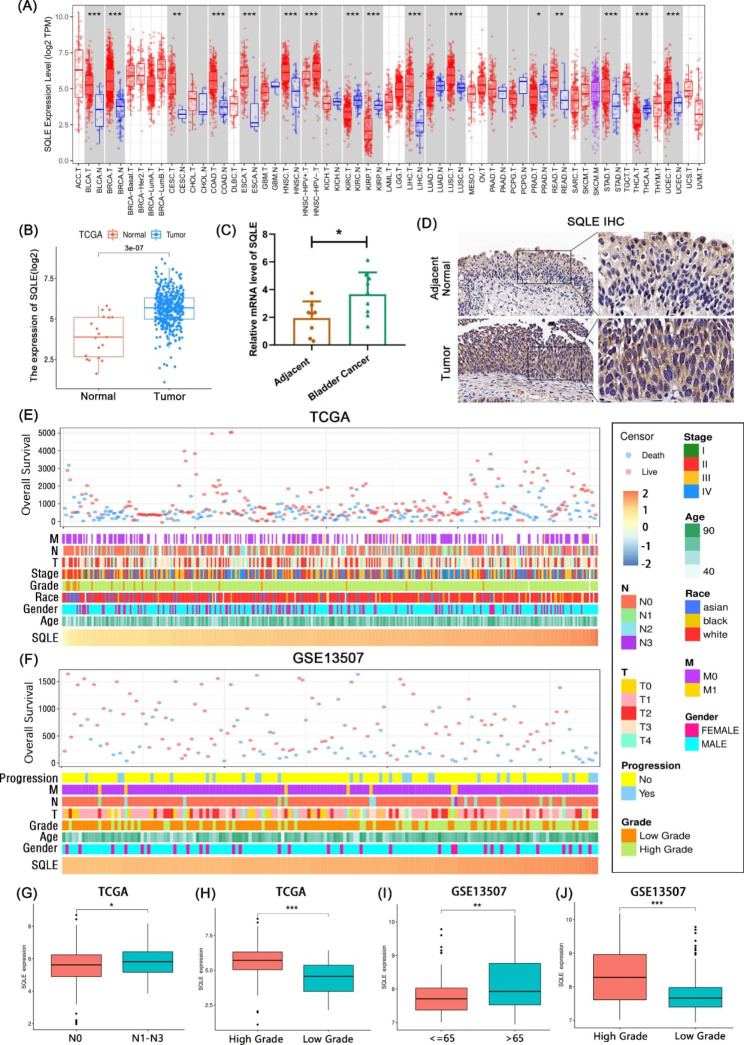
SQLE is highly expressed in BCa tissues and SQLE expression is associated with various clinical characters. **(A)** The mRNA expression levels of SQLE in 33 types of tumor tissues and adjacent normal tissues from the TIMER database; **(B)** The differential expression of SQLE between unpaired BCa tissues and adjacent normal tissues. The nucleus is blue and the brownish-yellow stain is positive; the darker the color, the higher the level of gene expression; **(C)** The differential mRNA expression of SQLE between bladder tumor tissue and adjacent normal tissue in BCa; **(D)** Representative immunohistochemistry of SQLE in BCa tissues and adjacent tissues. The nucleus is blue and the brownish-yellow stain is positive; the darker the color, the higher the level of gene expression; **(E)** The landscape of SQLE-related clinicopathological features of BCa in TCGA database; **(F)** The landscape of SQLE-related clinicopathological features of BCa in the GEO database; **(G–J)** The correlation between the expression of SQLE and some clinicopathological features of BCa. (*p < 0.05, **p < 0.01, *** *p* < 0.001)

### Quantitative real-time PCR

The total RNA from the clinical samples was extracted using TRIzol (Servicebio, Wuhan, China) following the instruction manual and then measured by NanoDrop 2000 (Thermo Fisher, USA). cDNA was synthesized by employing SweScript All-in-One First-Strand cDNA Synthesis SuperMix for qPCR (Servicebio, Wuhan, China) following the manufacturer’s protocol, and 2×Universal Blue SYBR Green qPCR Master Mix was used to amplify the cDNAs using the Lightcycler 4800II (Roche, Basel, Switzerland). A reference gene (GAPDH) was used to normalize the average CT value of target genes. We calculated the relative gene expression as 2^−ΔΔCt^. The sequences of primers (Sangon Biotech, Shanghai, China) were as follows:

GAPDH forward,5’-CAGGAGGCATTGCTGATGAT-3’,GAPDH reverse,5’-GAAGGCTGGGGCTCATTT-3’,

SQLE forward,5’-CTGACCTTTATGATGATGCAGC-3’and reverse,5’-CAGGCTTTTCTTAGTTGATGCA-3’.

### Immunohistochemistry assay

SQLE protein expressions in BCa and adjacent normal tissues were identified using an immunohistochemistry assay. First, fresh tumors were fixed in 4% paraformaldehyde (Biosharp, Beijing, China) for 24 h. Then, they were embedded in paraffin, and cut into 5-µm sections. Next, the sections were immunohistochemically stained by SQLE antibody (1:100). The DAB (Vector Laboratories) chromogen was used to incubate, and then hematoxylin was used to counterstain. Finally, the sections were analyzed under a light microscope (BX51, Olympus, Japan).

### Western blotting

Cell samples were lysed in the RIPA lysis buffer (Beyotimem, Shanghai, China) on the ice with 1% PMSF (Servicebio, Wuhan, China) and phosphatase inhibitor (Servicebio, Shanghai, China). Next, the lysates were centrifuged right away at 13,000 rpm for 15 min after being incubated on ice for 30 min. The supernatant from each sample was then collected, and the BCA assay was used to detect the concentration of sample protein. Then a 25% volume 5X Native Gel Sample Loading Buffer (Biosharp, Beijing, China) was added into the samples and boiled for 15 min at 100 °C. All protein samples were preserved at − 80 °C or − 20 °C for different types of storage. Equivalent protein from each sample was separated by 10% SDS-PAGE for 2 h at 80 V, and then protein was transferred to PVDF membranes (Millipore, NJ, USA) at a current of 200 mA. The membranes were then blocked with a protein-free rapid blocking buffer (Epizyme, Shanghai, China) for 15 min at room temperature. The membranes were then incubated with various primary antibodies overnight at 4 °C, followed by incubation with goat anti-mouse IgG (SA00001-1, Proteintech) or goat anti-rabbit IgG (SA00001-2, Proteintech) at room temperature for 1 h. Specific bands were tested with an ECL kit (Beyotime, Shanghai, China). Finally, a ChemiDoc™ Touch Imaging System (Bio-Rad) was used to scan protein bands and acquire images, and ImageJ software was used to analyze the results.

**Fig. 2:**
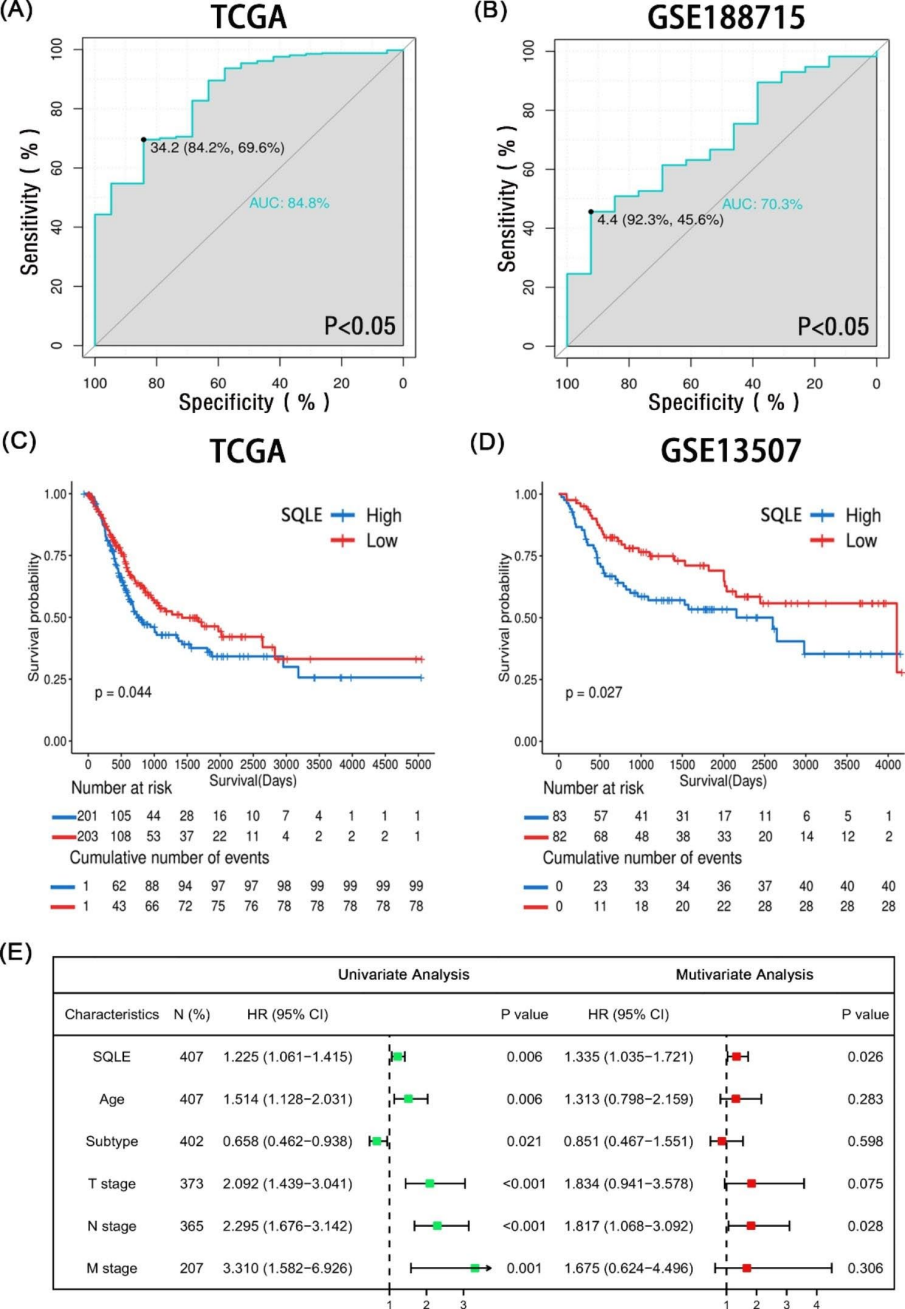
Comprehensive diagnostic and prognostic analyses of SQLE in BCa **(A, B)** The ROC curve indicated the high-expression specificity of SQLE in tumor and normal tissues in TCGA and GSE188715 databases; **(C, D)** Kaplan–Meier analysis of SQLE expression in TCGA and GSE13507 databases. The significance of the prognostic value was tested using a log-rank test; (E) Univariate and multivariate analyses of prognostic parameters in TCGA database overall survival (OS)

### Lentivirus and siRNA infection

The SQLE-si1: 5’-GCUCAGGCUCUUUAUGAAUUATT-3’, SQLE-si2: 5’-CCAGUUCUCAUCUACCAGAUUTT-3’, SQLE-si3: 5’-CCUGCCUUUCAUUGGCUUCUUTT-3’, and siNC: 5’-UUCUCCGAACGUGUCACGUTT-3’ were purchased from Sangon Biotech (Shanghai, China). The siRNAs were transfected into cells with the Opti-MEM culture medium (Gbico, US) and Lipofectamine 2000 (Invitrogen).

The lentiviral shRNA expression vectors for human SQLE: 5’-CCAGTTCTCATCTACCAGATT-3’ and control: 5’-CCTAAGGTTAAGTCGCCCTCG-3’ were also purchased from OBIO Scientific Service (Shanghai, China). The lentiviral shRNA expression vectors were transfected into cells with 6 µg/ml polybrene. After transfection for two days, the infected cells were selected by treatment with 2 µg/ml puromycin.

### CCK-8 assay

Cell viability was analyzed by Cell Counting Kit-8 (CCK-8, Biosharp, Shanghai, China) in accordance with the CCK8 assay protocols. Cells were seeded at a density of 2 × 10^3^/well in 100 µL of medium into 96-well plates (Corning, USA). Subsequently, in 24, 48, and 72 h, 10 µl of CCK-8 was added to the cells and incubated at 37 °C for 1 h. A microplate reader (Bio-Rad Laboratories, Inc.) was used to measure the absorbance of each well at 450 nm. Three independent experiments were conducted in each group.

### Colony-forming assay

T24 and 5637 cells were plated into six-well plates with the density of 500 cells per well for a 14-day incubation period. After fixation in methanol and staining with 0.5% crystal violet, colony formation rate was determined.

**Fig. 3:**
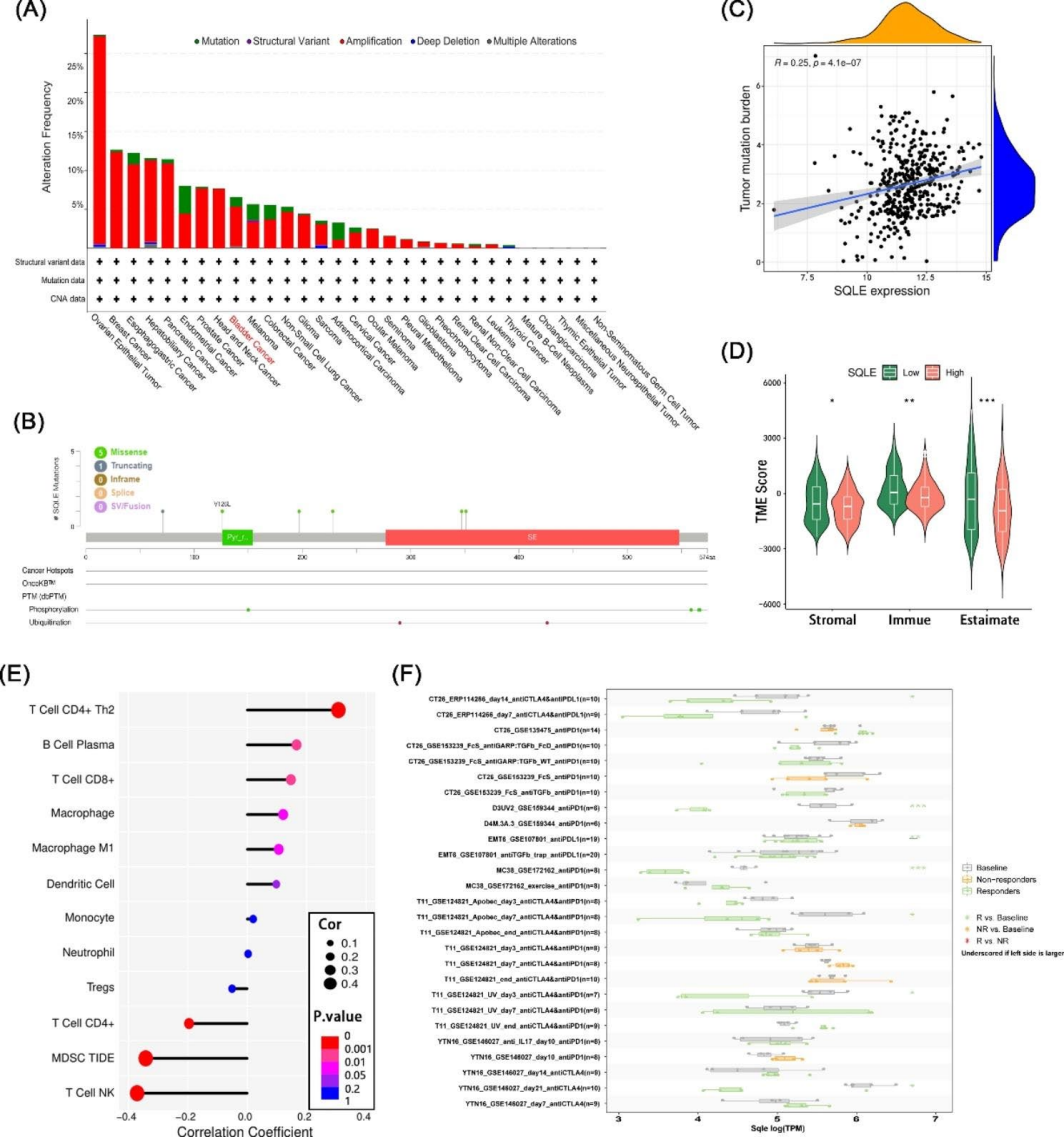
Alteration of SQLE and the association between SQLE expression and immune cell infiltration in BCa. **(A)** Alteration frequency of SQLE in BCa and pan-cancer. **(B)** The mutation sites, types of mutation, and alteration frequency of SQLE somatic mutation in BCa. **(C)** The correlation between tumor mutation burden and expression of SQLE. **(D)** The stromal, immune, and ESTIMATE scores were different between low-expression SQLE and high-expression SQLE. **(E)** SQLE expression was positively associated with CD4 + Th2 T cells, plasma cells, CD8 + T cells, and macrophage infiltration in BCa and negatively correlated with CD4 + T cells, myeloid-derived suppressor cells, and NK cells. **(F)** Gene expression after ICB treatment and in responders and non-responders in different tumor models. (*p < 0.05, **p < 0.01, ***p < 0.001)

### Immunofluorescence

Cells were grown on glass slides, and after reaching 70% confluency, the medium was thrown away and cells were washed with PBS, and then fixed with 4% paraformaldehyde for 15 min. Subsequently, after permeabilization with 0.5% Triton-X-100 for 15 min, cells were blocked with goat serum for 30 min at 4 °C, followed by incubation with primary antibodies at 4 °C overnight. After sufficient washing, cells were then incubated with fluorescence-conjugated secondary antibodies at room temperature for 1 h, followed by nuclear staining using DAPI. Images were acquired using a fluorescence microscope (BX53, Olympus, Japan).

### Wound-healing assay

After 48 h of transfection, T24 and 5637 cells were seeded in 6-well plates. Wounds were generated by scratching cell layer with 1-ml sterile plastic pipette tips and rinsed in the culture medium. The final images were acquired with a microscopy (IX51, Olympus, Japan).

**Fig. 4:**
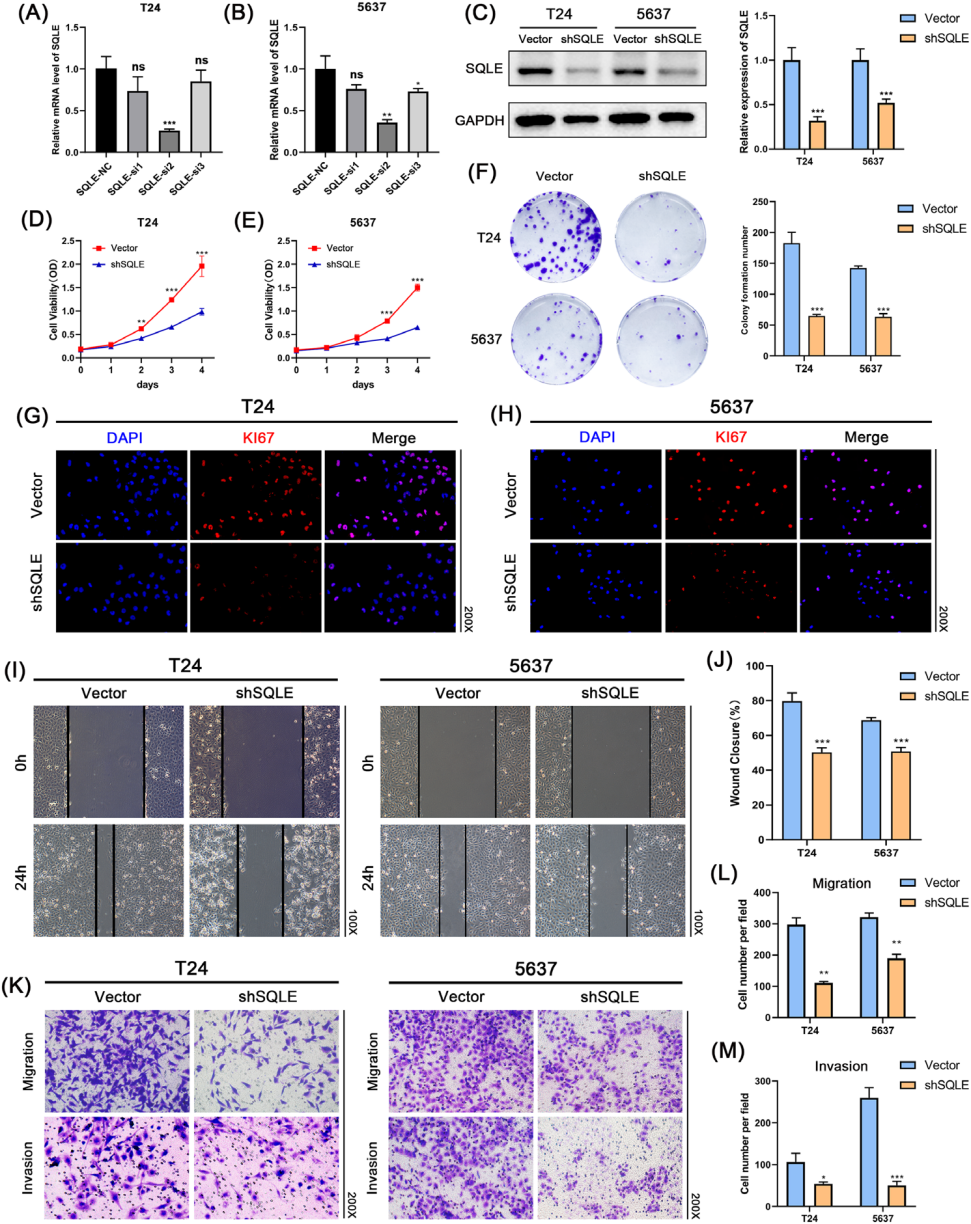
SQLE knockdown inhibited T24 and 5637 cell proliferation, migration, and viability. **(A, B)** The qRT-PCR assay detected shRNAs transfection efficiency in two BCa cell lines T24 and 5637; **(C)** Western blot assay detected the sh2 efficiency of transfection for protein level in two BCa cell lines T24 and 5637; **(D, E)** The CCK-8 assay was used to evaluate the proliferation of shSQLE-treated tumor cells and NC-treated tumor cells from 0 to 96 h; **(F)** Representative images of the colony-forming assay; **(G, H)** Immunofluorescence results showed that the expression of KI67 was decreased in BCa; **(I, J)** The wound-healing assay demonstrated that the capacity of migration in two BCa cell lines T24 and 5637 **(K–M)** The migration and invasion of T24 and 5637 cell lines were inhibited by SQLE knockdown as measured by a transwell assay. Data represent the mean ± standard deviation (SD) of three independent experiments. (The magnification under the microscope is shown as marked in the diagram; *p < 0.05, **p < 0.01, ***p < 0.001 versus the vector group, ns, not significant)

### Transwell migration and invasion assay

Cell invasion assay was performed with Matrigel-coated transwell chamber (Corning, NY, USA) following the standard method. To perform migration assays, 200 µl of serum-free medium containing 1 × 10^4^ T24 and 5637 cells were put to the upper chamber (8-mm pore size, Corning) with serum-free RPMI-1640, and 600 µl of complete medium was put into the lower chamber. The chamber was washed thrice with PBS after 48 h of incubation and was then fixed in 4% paraformaldehyde and stained with crystal violet. To perform the invasion assay, Matrigel and serum-free medium were first mixed at a ratio of 1:8. Then, 80 µl of the mixed solution was added to the upper chamber, and the remaining experimental steps were the same as the migration experiment. Five fields per well were randomly selected and observed under a microscope (IX71, Olympus, Japan).

### Flow cytometry analysis

For cell cycle analysis, transfected cells were reaped and fixed in 70% pre-iced ethanol at 4 °C for 10 min, followed by incubation with RNase and propidium iodide (PI; Yeasen, Shanghai, China). The proportions of cells in G0/G1, S or G2/M phases were determined using the Cell Quest Pro acquisition software (BD Biosciences, Franklin Lakes, NJ, USA). For cell apoptosis analysis, cells were double-stained with Annexin V-fluorescein isothiocyanate (FITC; BD Biosciences) and PI, tested using flow cytometry.

**Fig. 5:**
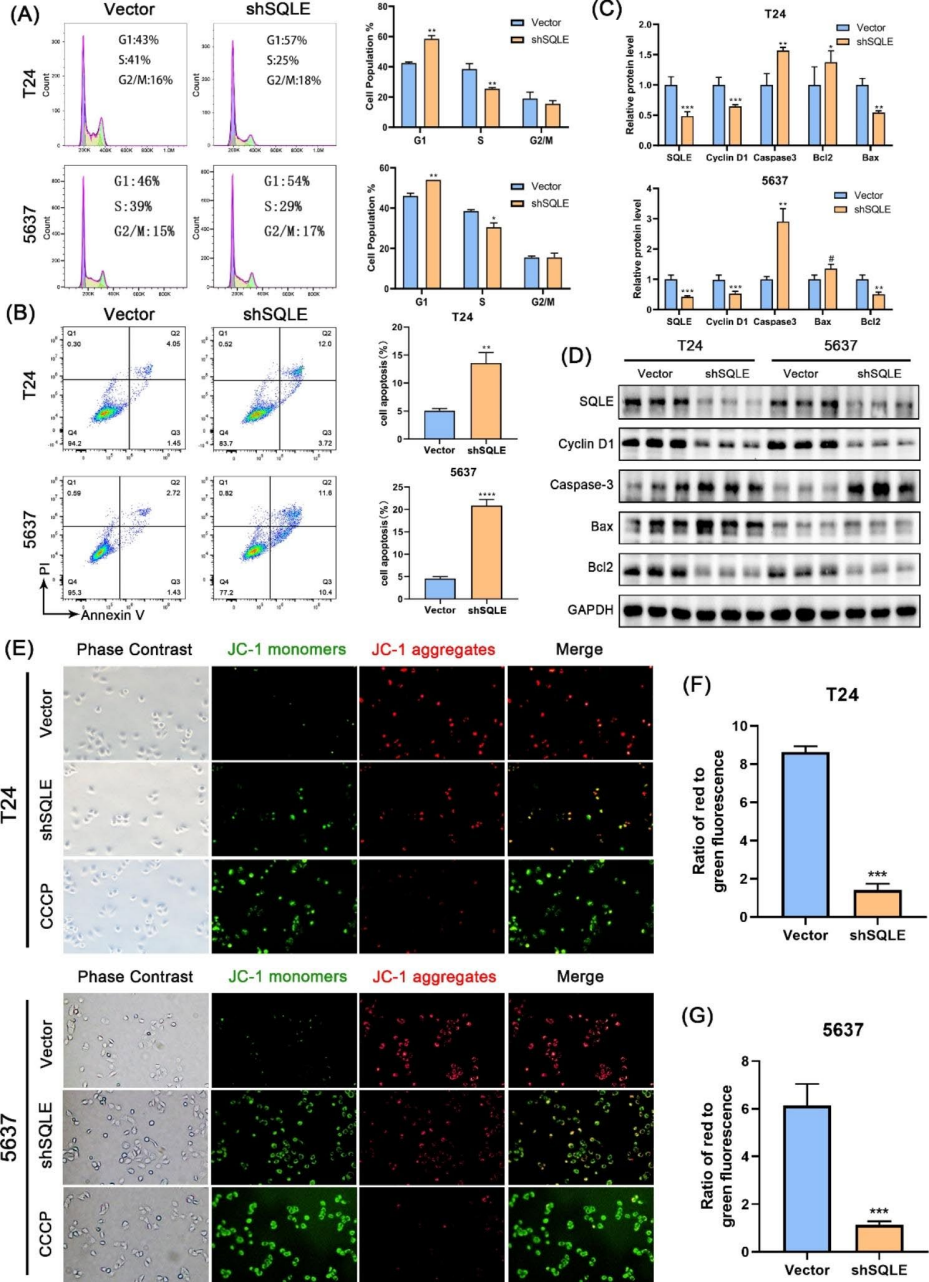
SQLE Knockdown inhibited T24 and 5637 cell cycle and promoted cell apoptosis. **(A)** Flow cytometry was used to detect the proportion of G1, S, and G2/M phase cell population in T24 and 5637; **(B)** Flow cytometry was used to detect the early apoptosis and late apoptosis cell population in T24 and 5637; **(C, D)** The western blot assay was used to evaluate the expression of cell cycle and cell apoptosis related genes; **(E–G)** Representative images of JC-1 immunofluorescence and the ratio of red to green fluorescence showed a decrease in the mitochondrial membrane potential. Data are presented herein as the from three independent experiments. Data represent the mean ± SD of three independent experiments. (*p < 0.05, **p < 0.01, ***p < 0.001 versus the vector group, ns, not significant)

### Animal experiments

Four-week-old Balb/c nude mice were purchased from the Centre of Experimental Animals at Wuhan University Medicine College (Hubei, China). T24 cells (5 × 10^6^) stably transfected with SQLE knockout lentivirus (shSQLE) or control lentivirus (shNC) were subcutaneously injected into each mouse. One week after the injection of tumor cells, mice in the drug-treated group were given perfusion of PFT-β hydrobromide into the stomach of mice every week (2.5 mg/kg). In the remaining groups without PFT-β hydrobromide intervention, the mice were administered an isovolumetric injection of normal saline. The growth of tumor size (L, longest dimension; W, shortest dimension) was monitored by the caliper rule every five days, and tumor volumes were calculated using the formula: V = L × W × W/2 [20]. The tumors were subsequently harvested for further research.

### Statistical analyses

SPSS Statistics 26 software was used for all statistical analyses. All data are expressed as the mean ± SD. Student’s t test was used for comparisons between different groups. The X^2^ test was used to assess the correlation between the level of protein and clinical characteristics. Spearman’s correlation analysis was used to evaluate the relationship between target genes. A p-value < 0.05 was considered statistically significant.

**Fig. 6:**
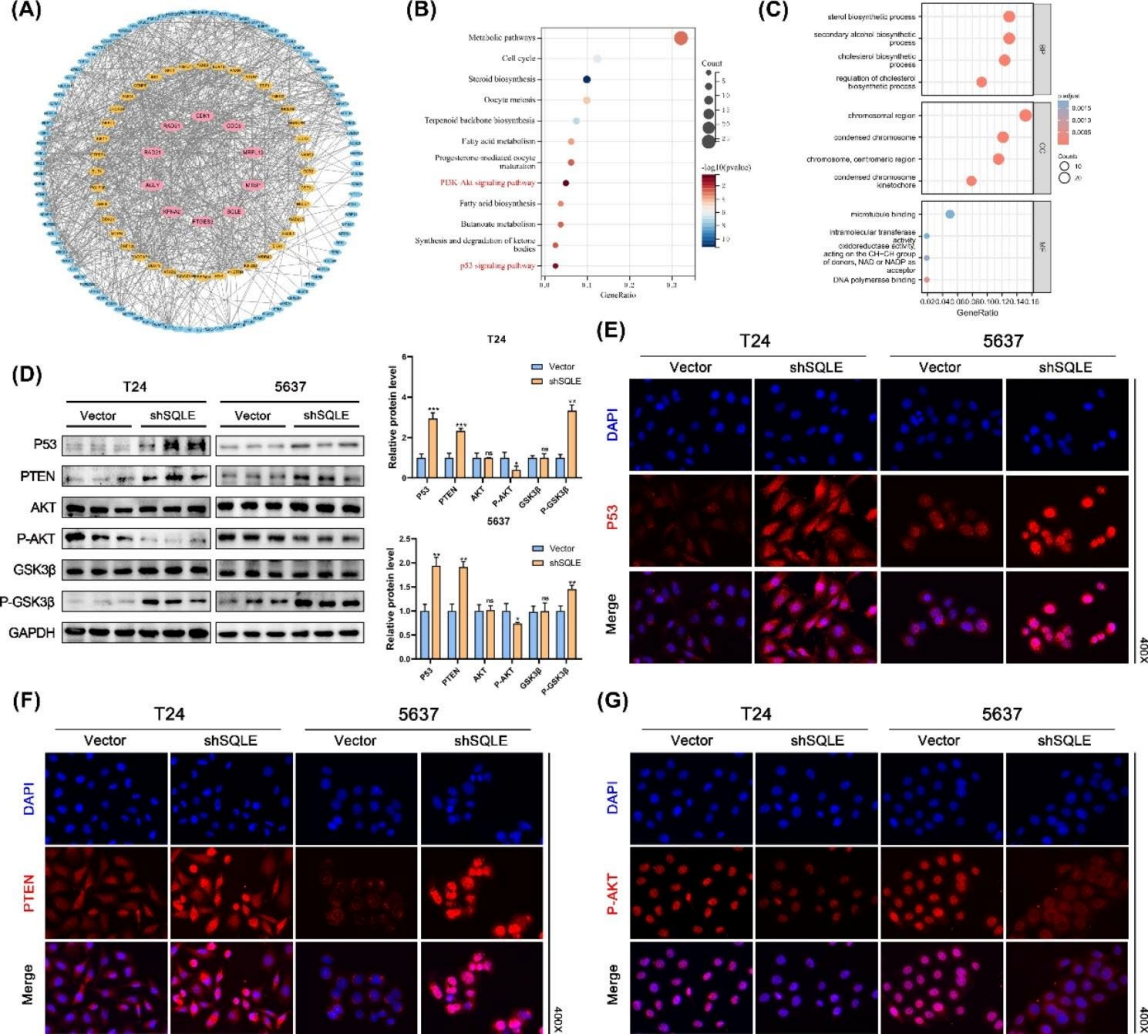
Knockdown of SQLE affects the P53 signaling pathway and PTEN/AKT/GSK3β signaling pathway. **(A)** The top 147 genes associated with SQLE were constructed with a PPI network (23 genes were not found in the STRING platform); **(B)** KEGG enrichment analysis of genes in PPI network; **(C)** GO enrichment analysis of genes in PPI network; **(D)** Protein expression levels of P53, PTEN, AKT, P-AKT, GSK3β, and P-GSK3β in T24 and 5637 cells; **(E–G)** Representative images of P53, PTEN, and P-AKT immunostaining. Data represent the mean ± SD of three independent experiments. (*p < 0.05, **p < 0.01, ***p < 0.001 versus the vector group, ns, not significant)

## Result

### SQLE is highly expressed in BCa as well as in many other cancers

First, TIMER 2.0 was used to study the expression level of SQLE in pan-cancer. The results revealed that SQLE was upregulated in pan-cancer (Fig. 1A). Meanwhile, TCGA and GSE188715 databases were used to demonstrate that, compared with the normal bladder epithelial tissues, the transcription level of SQLE is highly expressed in paired and unpaired BCa tissues (Fig. 1B; Figures S1A–S1C). Then we selected nine BCa tissues and eight adjacent normal tissues for qRT-PCR assays (Fig. 1C). In addition, five paired tumor tissues with significant differences and adjacent normal tissues were used for immunohistochemical staining (Fig. 1D). The results demonstrated that SQLE expression was significantly different between tumor tissue and adjacent normal tissue in BCa, both at the mRNA and protein levels.

Patients with various expression levels of SQLE similarly have different clinical and pathological characters, such as clinical stage, WHO group, race and region, age, disease progression, gender, overall survival, and vital status in TCGA and GSE13507 databases (Fig. 1E F). By further analysis, we found that SQLE was highly enriched in pathological classification of the N1–N3 stage than N0 stage in BCa (Fig. 1G), and different ethnic groups also expressed SQLE at different levels in BCa, with black ethnic group having the highest SQLE expression (Figure S1C). Furthermore, high-grade BCa usually presented a higher SQLE expression (Fig. 1H J). In the GSE13507 database, BCa patients older than 65 years have a higher SQLE expression (Fig. 1I), and other data of SQLE in TCGA are depicted in supplementary Figure S1. In summary, our results indicate that the high expression of SQLE, as an oncogene, can promote the progression of BCa and indicate the malignancy of BCa.

**Fig. 7:**
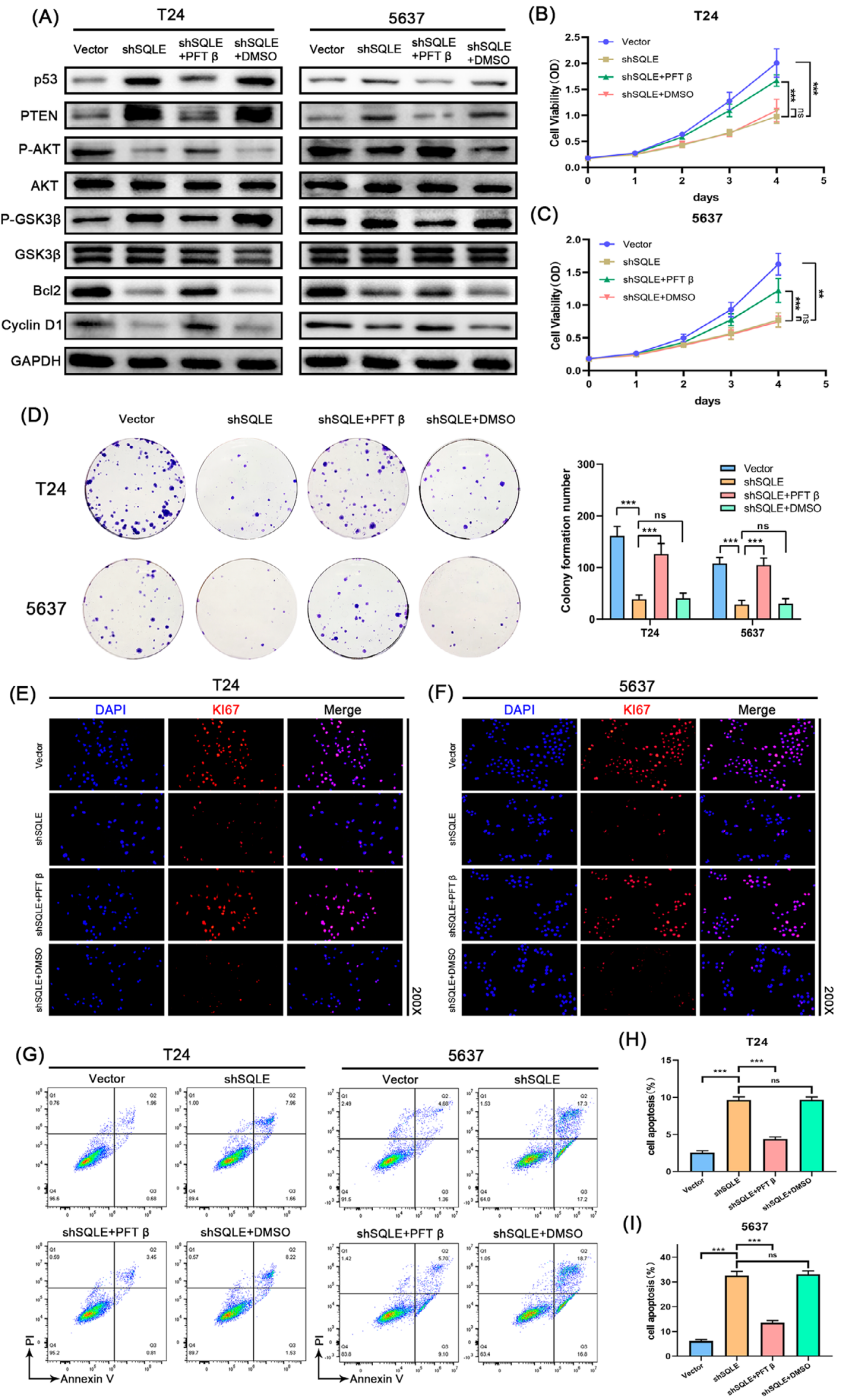
SQLE increases tumor cell proliferation and inhibits cell apoptosis of Bca by regulating the PTEN/AKT/GSK3β signaling pathway through P53. **(A)** Protein expression levels of P53, PTEN, AKT, P-AKT, GSK3β, P-GSK3β, Bcl2, and Cyclin D1 in T24 and 5637 cells; **(B, C)** The CCK-8 assay was used to assess the proliferation of shSQLE-treated tumor cells, NC-treated tumor cells, negative controls, and cells treated with shSQLE + PFT β from 0 to 96 h; **(D)** Representative images of the colony-forming assay; **(E, F)** Representative images of KI67 immunostaining showed that overexpression of P53 increased the proliferation of T24 and 5637 cells after SQLE knockdown; **(G–I)** Flow cytometry detected the early apoptosis and late apoptosis cell population in T24 and 5637. Data represent the mean ± SD of three independent experiments (*p < 0.05, **p < 0.01, ***p < 0.001, ns, not significant)

### Diagnostic and prognostic values of SQLE in BCa

To completely understand the diagnostic function of SQLE in BCa, TCGA and GSE188715 cohort databases were selected for this study. The receiver operating characteristic (ROC) curves indicated that SQLE plays a key role in BCa diagnosis (Fig. 2A and B), especially in TCGA database the area under the curve (AUC) value reached 84.8% (p < 0.05). We did not include GSE188715 in the plotting of the ROC curve owing to the small number of normal samples. Consequently, SQLE the expression of can also be used as a potential diagnostic indicator for likelihood of BCa.

We next attempted to verify whether the differential expression of SQLE will influence patients’ prognosis in BCa. By the level of median SQLE expression, we classified patients in TCGA and GSE13507 databases into high- and low-risk groups and found that survival probability was significantly lower in the high-risk group (Fig. 2C and D). Furthermore, we found that SQLE expression was a prognostic factor, independent of known prognostic factors, in the univariate and multivariate Cox regression analyses (Fig. 2E), including age at diagnosis, pathological subtypes, T stage, N stage, and M stage. The above results indicate that the expression level of SQLE, a potential new biomarker, has implications for the diagnosis and prognosis of BCa.

**Fig. 8:**
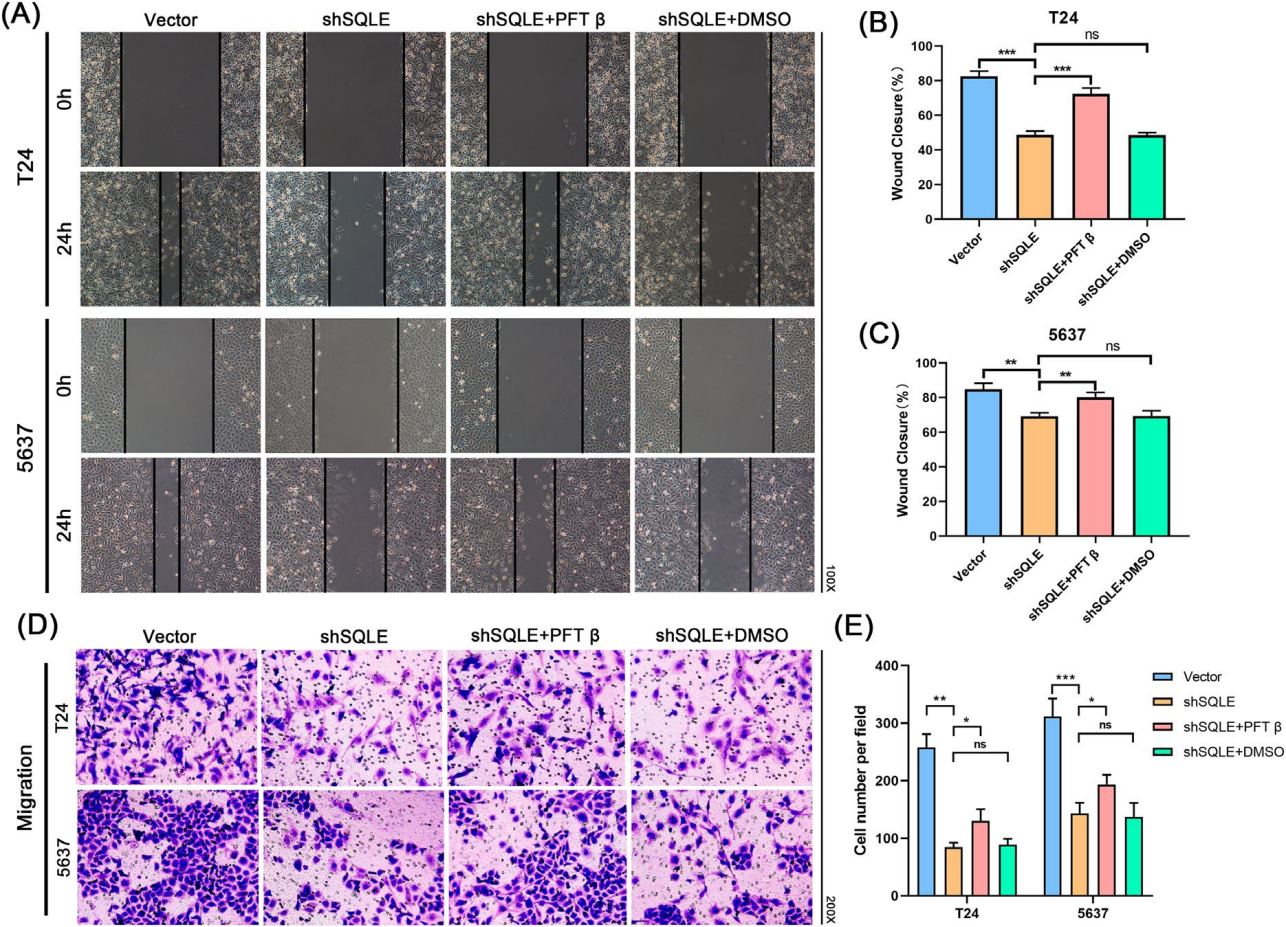
SQLE increases tumor cell migration and invasion by regulating the PTEN/AKT/GSK3β signaling pathway through P53. **(A–C)** The wound-healing assay demonstrated the capacity of migration in two BCa cells T24 and 5637; **(D, E)** The migration of T24 and 5637 cells were rescued by upregulation of P53 as measured by a transwell assay; Data represent the mean ± SD of three independent experiments. (*p < 0.05, **p < 0.01, ***p < 0.001, ns, not significant)

### SQLE is susceptible to mutation and is related to immune infiltration in BCa

The cBio Cancer Genomics Portal (cBioPortal) was used to explore the potential role of SQLE in alteration status. We noted that SQLE was widely altered in pan-cancer, and in BCa, the alteration frequency approximately reached 7% (Fig. 3A). Furthermore, we explored alteration sites, types, and numbers of SQLE (Fig. 3B). A correlation scatter plot was plotted to illustrate the relationship between the expression of SQLE and gene mutations in BCa (Fig. 3C).

Tumor microenvironment (TME) – the environment around a tumor – can cause tumor development [21, 22] and contribute to the evasion of the immune system by tumor cells [23, 24]. We scored a wide range of cells infiltrating the TME. The results revealed that the stromal score, immune score, and ESTIMATE score were significantly correlated with SQLE expression in BCa (Fig. 3D). CIBERSORT, TIMER, TIDE, and xCELL databases were used to investigate the correlation between SQLE expression and immune infiltration. We observed that SQLE was positively correlated with CD4 + Th2 T cells, plasma cells, CD8 + T cells, and macrophages and negatively correlated with CD4 + T cells, myeloid-derived suppressor cells and NK cells (Fig. 3E). These results demonstrated that SQLE could be a molecular regulator of immune cell infiltration in TME. After comparing the gene expression before and after immune checkpoint blockade (ICB) treatment in different tumor models and between responders and non-responders, we found that SQLE expression was closely related to the effectiveness of ICB treatment (Fig. 3F).

**Fig. 9:**
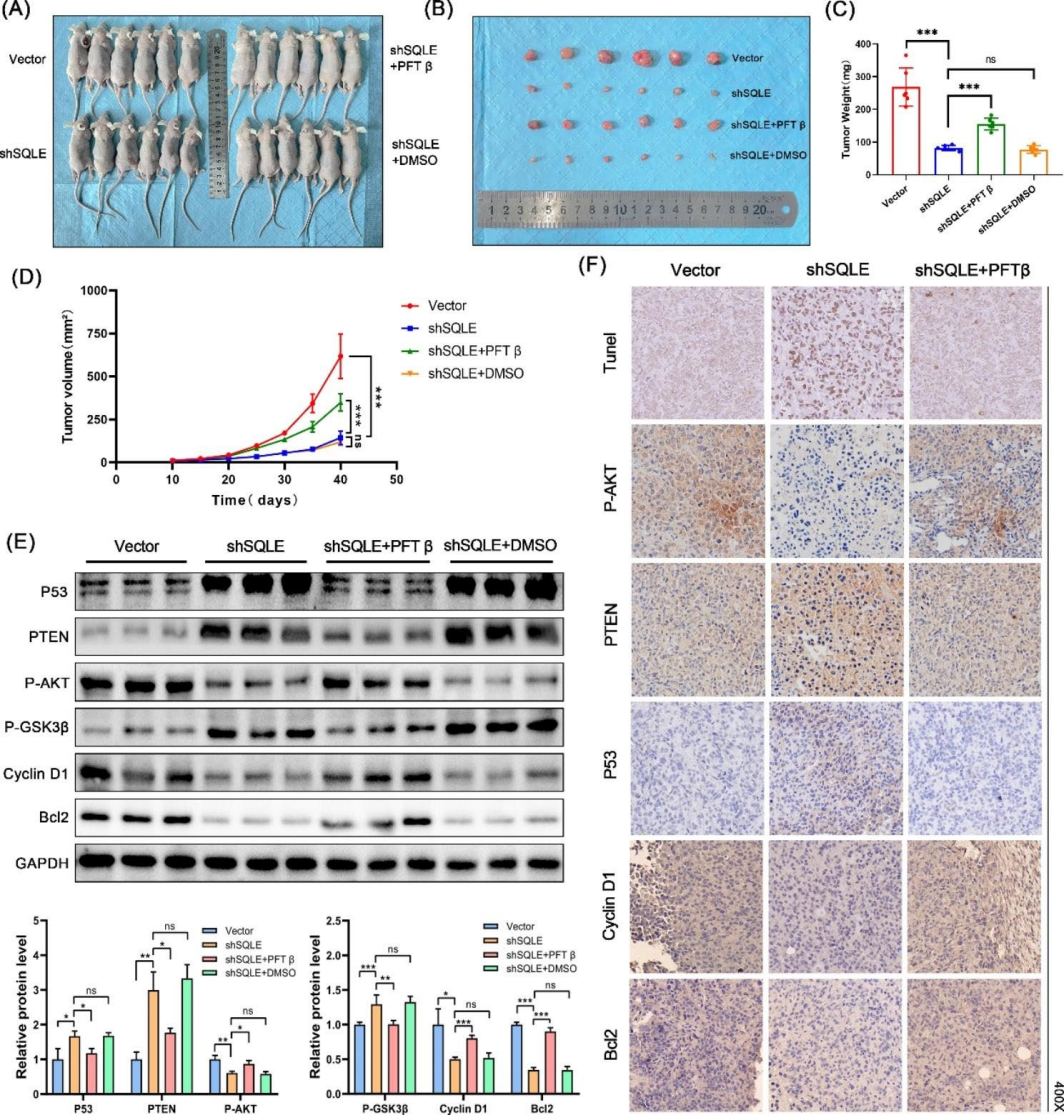
SQLE increases tumor cell proliferation, migration, and invasion by regulating the PTEN/AKT/GSK3β signaling pathway through P53 in vivo. **(A–B)** Representative image of T24 xenograft tumors under different treatments; **(C)** Tumor weight in each group; **(D)** Tumor volume in each group; **(E)** Expression levels of P53, PTEN, P-AKT, P-GSK3β, cyclin D1, and Bcl2 of xenograft tumors in each group as determined by the western blot assay; **(F)** Expression of P-AKT, PTEN, P53, Cyclin D1 and Bcl2 in xenograft tumors was analyzed by immunohistochemical staining and TUNEL staining assays. The nucleus is blue and the brownish-yellow stain is positive; the darker the color, the higher the level of gene expression. Data represent the mean ± SD of three independent experiments. (*p < 0.05, **p < 0.01, ***p < 0.001, ns, not significant)

### SQLE knockdown reduced BCa cell proliferation and migration in vitro

To assess the functions of SQLE in BCa, three independent siRNAs for SQLE were transfected into T24 and 5637 cell lines to construct two stable cell lines. By using the qRT-PCR assay (Fig. 4A and B), the transfection efficiency of three siRNAs was verified, and si2 was chosen for further experiments. In two BCa cell lines T24 and 5637, western blot analysis confirmed the same results for protein level (Fig. 4C). Furthermore, the CCK-8 assay demonstrated that SQLE knockdown decreased BCa cell proliferation ability in T24 and 5637 cell lines (Fig. 4D and E). The colony-forming assay was used to assess the proliferation ability of a single tumor cell. The results revealed that SQLE depletion could inhibit the proliferation of BCa cells (Fig. 4F). As is known, KI67 is closely related to cancer proliferation and can be treated as a prognostic and predictive marker [25, 26]. Results of immunofluorescence revealed that SQLE knockdown significantly inhibited the expression of KI67 in BCa cell lines (Fig. 4G H). The wound-healing assay and transwell migration assay were used to detect the migratory and invasive ability of tumor cells. Both assays demonstrated that SQLE knockdown suppressed cell migration and invasion in T24 and 5637 cell lines (Fig. 4I M).

**Fig. 10:**
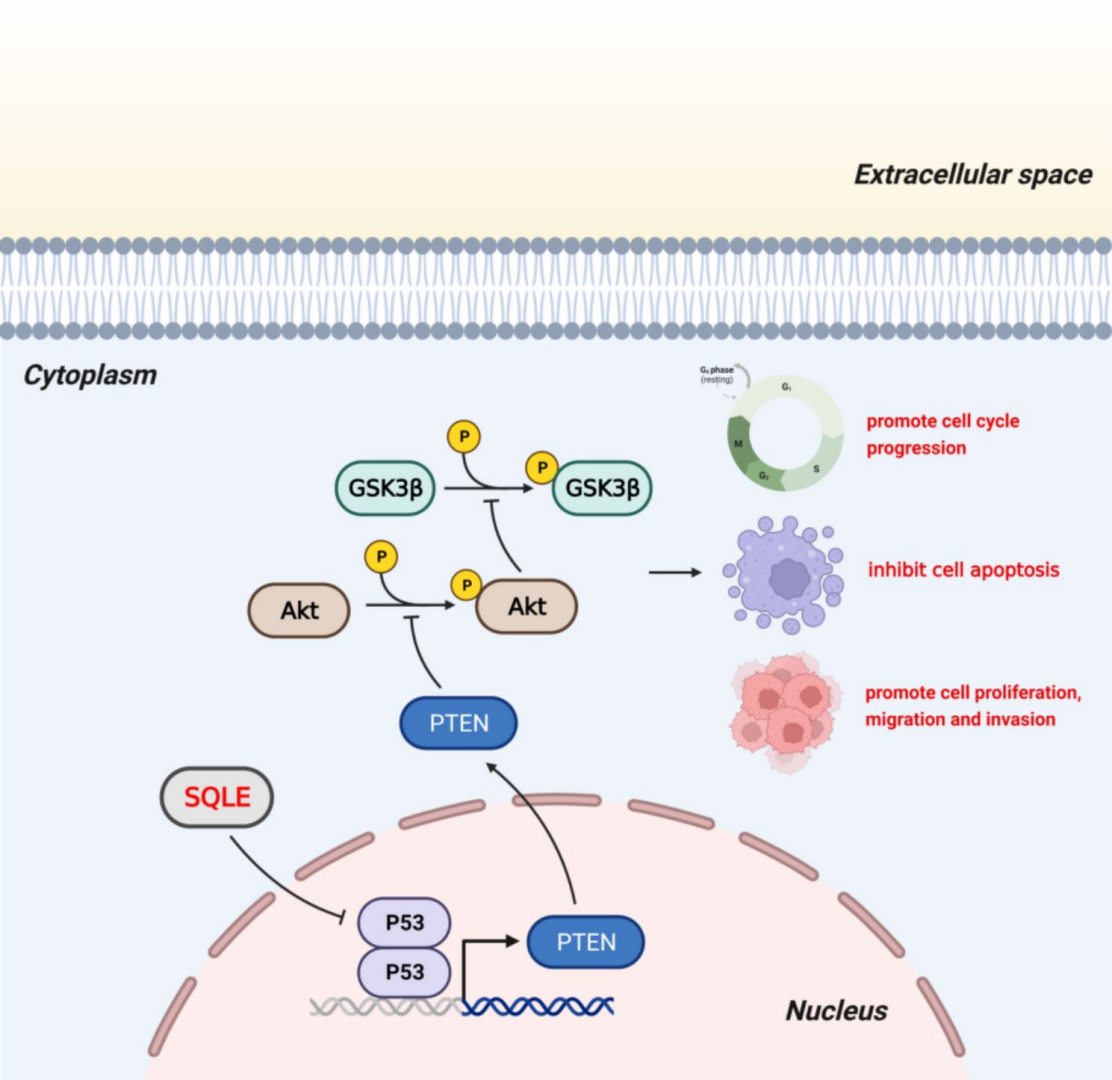
This schematic diagram explains the molecular mechanisms underlying the pro-BCa effects of SQLE. Briefly, SQLE regulates the expression of downstream PTEN/AKT/GSK3β through P53 molecules, thereby promoting BCa proliferation, migration, and invasion and inhibiting apoptosis

### SQLE downregulation suppressed cell cycle progression and promoted apoptosis in vitro

Cell cycle is an essential process for tumor cell growth [27]. To investigate whether SQLE regulates cell growth by affecting cell cycle, flow cytometry was used to detect the proportion of cells in G1, S, and G2/M phases. The results revealed that SQLE knockdown at T24 and 5637 cell lines increased G1 phase cell number, with a concomitant decrease in S phase cells (Fig. 5A). However, the cells in G2/M phase population did not change significantly. Western blot showed that cyclin D1 decreased in SQLE knockdown cell lines (Fig. 5C and D), which also indicated that SQLE promoted cell cycle progression in BCa. Meanwhile, flow cytometry similarly verified that compared with the vector group, SQLE knockdown increased cell apoptosis in T24 and 5637 cell lines (Fig. 5B). Western blot results revealed that SQLE knockdown further resulted in an increase in the apoptosis-promoting genes caspase-3 and Bax and a decrease in the apoptosis-suppressing gene Bcl2 (Fig. 5C and D). The level of mitochondrial membrane potential is an important predictor of early apoptosis [28]. In the present study, we used JC-1 stain to detect mitochondrial membrane potential levels. The results showed that SQLE knockdown, with a significant decrease in green fluorescence intensity, was accompanied by an increase in red fluorescence intensity (Fig. 5E and G), indicating that SQLE knockdown significantly reduced mitochondrial membrane potential levels. The above results illustrate that SQLE plays an important role in the progress of BCa by regulating the process of cell cycle and apoptosis.

### SQLE affects the P53 and PTEN/AKT/GSK-3β signaling pathways in vitro

To further explore the biological function of SQLE, TCGA database was utilized to identify the 147 genes most associated with SQLE in BCa, and STRING database and Cytoscape were used to construct a PPI network (Fig. 6A). Twenty-three genes of them were not shown in the PPI network. Furthermore, we extracted 170 genes most associated with SQLE for KEGG and GO enrichment analysis (Fig. 6B C). The KEGG results showed that SQLE plays a vital role in lipid metabolism, cell cycle, PI3K/AKT signaling pathway, and P53 signaling pathway, and the GO results showed that SQLE is associated with cholesterol metabolism, chromosome-related functions, microtubule binding and DNA polymerase binding. Next, western blotting was used to detect the expression levels of proteins in BCa, including P53, PTEN, AKT, P-AKT, and their respective phosphorylated forms (Fig. 6D). The results showed that SQLE silencing resulted in a substantial increase in P53, PTEN, and phosphorylated GSK3β, while phosphorylated AKT was remarkably decreased in T24 and 5637 cell lines. Immunofluorescence of P53, PTEN, and P-AKT further confirmed this result (Fig. 6E and G). The PTEN/AKT/GSK3β signaling, as a central anti-apoptotic intracellular signaling pathway, regulated cell growth, survival, proliferation, differentiation, and migration [29, 30]. In brief, we demonstrate herein that SQLE affects the P53 signaling pathway and PTEN/AKT/GSK3β signaling pathway in BCa, which may be the key pathway for SQLE to regulate the progress of Bca.

### PFT-β hydrobromide rescues the anti-BC effect of lentivirus knockdown of SQLE in vitro

To further demonstrate that SQLE exerts pro-tumor effects via the P53 signaling pathway and the PTEN/AKT/GSK3β signaling pathway, we first selected PFT-β hydrobromide (PFT-β), an inhibitor of the p53 protein, for rescue experiments. Western blotting analysis revealed that rescue of P53 activity led to a significant decrease in PTEN expression levels, which in turn affected the AKT signaling pathway (Fig. 7A, Figure S2A-D). The results showed that the regulation of SQLE knockdown on downstream PTEN/AKT/GSK3β pathway was reversed after inhibiting P53. Then the CCK-8 assays, colony-forming assays, and immunofluorescence assays were used to confirm that the proliferation capacity of T24 and 5637 cells were reversed (Fig. 7B F) after a reduction in P53 expression. Flow cytometry showed that the downregulation of P53 inhibited apoptosis in response to SQLE knockdown (Fig. 7G and I), which promoted BCa cell proliferation.

Furthermore, wound-healing and transwell assays were utilized to verify whether SQLE affected cell migration via the P53 and PTEN/AKT/GSK3β signaling pathways. The results showed that overexpression of P53 increased the migration of T24 and 5637 cells after SQLE knockdown (Fig. 8A and E). Western blotting analysis confirmed that the expression level of P53 in the shSQLE + PFT β group was significantly downregulated compared with the shSQLE group in BCa cells (Fig. 8F and G). Meanwhile, the western blot analysis also showed that the activity of rescuing P53 resulted in a significant decrease in PTEN expression level in the shSQLE + PFT β group compared with the shSQLE group, while the expression levels of P-AKT was significantly upregulated in BCa cells (Fig. 7A, Figure S2A-D). Overall, these results indicate that SQLE can inhibit BCa progression via the PTEN/AKT/GSK3β signaling pathway through P53.

### PFT-β hydrobromide rescues the anti-BC effect of lentivirus knockdown of SQLE in a tumor-bearing nude mouse model

To further explore SQLE regulation of cell proliferation through the P53/PTEN/AKT/GSK3β axis, we established a T24 xenograft tumor model in nude mice. We found that SQLE knockdown inhibited tumor growth and PFT-β hydrobromide could reverse the inhibitory effect of SQLE accordingly (Fig. 9A and D). Meanwhile, western blotting analysis revealed that SQLE knockdown upregulated P53 and PTEN and downregulated P-AKT, P-GSK3β, cyclin D1, and Bcl2 in vivo (Fig. 9E). After injecting P53 inhibitors into nude mice, we found that the expressions of P53, PTEN, and P-GSK3β decreased significantly, while the expressions of P-AKT, cyclin D1, and Bcl2 increased significantly. The results demonstrated that SQLE could promote bladder cancer proliferation and inhibit apoptosis and cell cycle arrest, and this effect is mediated by regulating the P53/PTEN/AKT/GSK3β axis in vivo. TUNEL staining assays and immunohistochemical staining provided us with further evidence of this conclusion (Fig. 9F). This result is also consistent with our previous in vitro results.

## Discussion

Recent studies [1] have shown that BCa is the 12th most common malignancy worldwide, second only to prostate cancer in terms of new cases of urological tumors in 2020 and accounting for nearly 3% of all new diagnoses [31, 32]. Furthermore, the majority of BCa cases originate from the uroepithelium [33]. Recent improvements in the surgical approach and improvements in the standard of surgeons have greatly improved the survival rate of BCa patients. However, there is still a lack of less invasive and more precise adjuvant treatments to improve the prognosis of patients. Metabolism is a fundamental biologically active feature of living organisms and is primarily associated with the uptake of substances and energy [34]. Previous studies showed that in addition to storing and supplying energy and participating in the composition of biological membranes, lipids are also involved in intercellular information transfer [35, 36], which play an important role in regulating the proliferation of cancer cells. SQLE, as a key rate-limiting enzyme, is a member of this group [37, 38]. In the present study, we found that SQLE promoted the proliferation and metastasis of BCa by regulating P53 through the PTEN/AKT/GSK3β signaling pathway.

The rapid growth of tumor cells is mainly ascribable to the disorder of the cell cycle, which consists of the S phase of DNA synthesis, the M phase of cell division, and the G1 and G2 intervals. Disruptions in any of these phases can cause abnormal cell proliferation [39, 40]. We found that after downregulation of SQLE in BCa cells, more cells were blocked in the G1 phase and did not enter the S phase. Cyclin D1, a subtype of cyclin, forms complexes with CDK4 or CDK6 and acts as their regulatory subunit, two cyclin-dependent kinases that are essential for the G1-to-S phase transition [41, 42]. This is why we were able to observe changes in cyclin D1 at the protein level. It is worth noting that the mechanism of action of SQLE in promoting bladder cancer cell proliferation described herein is different from that of hepatocellular carcinoma and colon cancer. Liu et al. [16] found that SQLE silences PTEN in hepatocellular carcinoma through ROS-mediated DNA hypermethylation, whereas we found that SQLE also silences PTEN through direct regulation of downstream P53. In brief, the mechanism of SQLE in BCa is complex, and our study provides a new idea for the treatment of BCa.

Apoptosis plays a key role in the evolution of organisms, in the stability of the internal environment and in the development of several systems. Tumorigenesis is closely linked to the inhibition of apoptosis, leading to the malignant transformation of affected cells into uncontrolled, indefinitely proliferating tumor cells [43, 44]. In addition to flow cytometry and apoptosis-associated protein assays, we examined mitochondrial membrane potential levels using the JC-1 probe and found that the downregulation of SQLE expression resulted in a decrease in the mitochondrial membrane potential. This additionally predicts that the presence of SQLE can organize the early apoptosis of BCa cells from the level of mitochondrial membrane potential.

P53, the single most important oncogene in cancer, is mutated in more than half of all human cancers [45]. It belongs to a larger gene family that includes two other highly related proteins, p63 and p73 [46, 47], and is involved as a key transcription factor encoding multiple molecules in the RNA sequence and thereby regulates downstream signaling pathways. Thus, numerous studies have targeted cancer therapeutic approaches to P53 [48, 49]. In the present study, we also found that SQLE can act as another important negative regulator upstream of P53 in T24 and 5637 cells. The PI3K/AKT signaling pathway begins with the activation of membrane receptor tyrosine kinases (RTK), which typically include the epidermal growth factor receptor (EGFR), fibroblast growth factor receptor (FGFR), and insulin-like growth factor I receptor [50] (IGF-IR), and this signaling pathway was involved in various biological functions such as glycolysis, apoptosis, and cell cycle [51, 52]. Studies have demonstrated [53, 54] that excessive activation of this signaling pathway can accelerate tumor cell proliferation, migration, and invasion, resulting in poor prognosis. By KEGG enrichment analysis, we found that SQLE was enriched in the P53 signaling pathway, and through experiments we verified that P53 acts as a mediator of SQLE regulation of the PTEN/AKT/GSK3β signaling pathway. This is in contrast to previous studies by Sun [55] and Liu [16], who only identified P53 as a gene that regulates cell proliferation, and our study identified a downstream pathway of P53 in BCa. This also reemphasizes the role of SQLE and P53 in the proliferation of BCa cells.

Previous studies [56] have shown that P53 can interact with PTEN as an upstream molecule of PTEN to regulate the AKT signaling pathway, but less has been reported in the literature. In the present study, we differed from the mechanisms of SQLE studied in other cancers by a rare association of two major oncogenes, P53 and PTEN, as their downstream molecules. The results showed that the proliferation, migratory blackness, and invasive ability of bladder cancer cells were restored after the re-inhibition of P53 in BCa cells knocked out of SQLE. In summary, overexpression of SQLE in BCa cells suppressed the expression of the oncogene P53, which in turn led to increased proliferation, migration, and invasion of BCa cells by affecting the level of PTEN and finally the aberrant activation of AKT. Upregulated SQLE is key to the prognosis of BCa patients. The detailed molecular mechanism is depicted in Fig. 10.

It is worth noting that the current study has a few limitations that should be addressed. Owing to difficulties in collecting clinical specimens, we selected only nine pairs of BCa and adjacent tissues for pre-testing. In addition, although we found that P53 can act as a downstream molecule of SQLE and thus regulate the PTEN/AKT/GSK3β signaling pathway, how the two molecules exert interactions was not explored further. This is a direction for our future research.

In conclusion, this study demonstrates that SQLE exerts pro-BCa effects in vivo and in vitro through P53 regulation of the PTEN/AKT/GSK3β signaling pathway, thus providing a new potential target for the treatment of BCa.

### Electronic supplementary material

Below is the link to the electronic supplementary material.

Supplementary Material 1

Supplementary Material 2

Supplementary Material 3

Supplementary Material 4

Supplementary Material 5

## Data Availability

All raw data is provided as required.
