# Supplementary figures and images for: SQLE Knockdown inhibits bladder cancer progression by regulating the PTEN/AKT/GSK3β signaling pathway through P53
Source: Cancer Cell Int. 2023 Sep 28;23:221. doi: 10.1186/s12935-023-02997-5 (PMC10540347; doi:10.1186/s12935-023-02997-5)

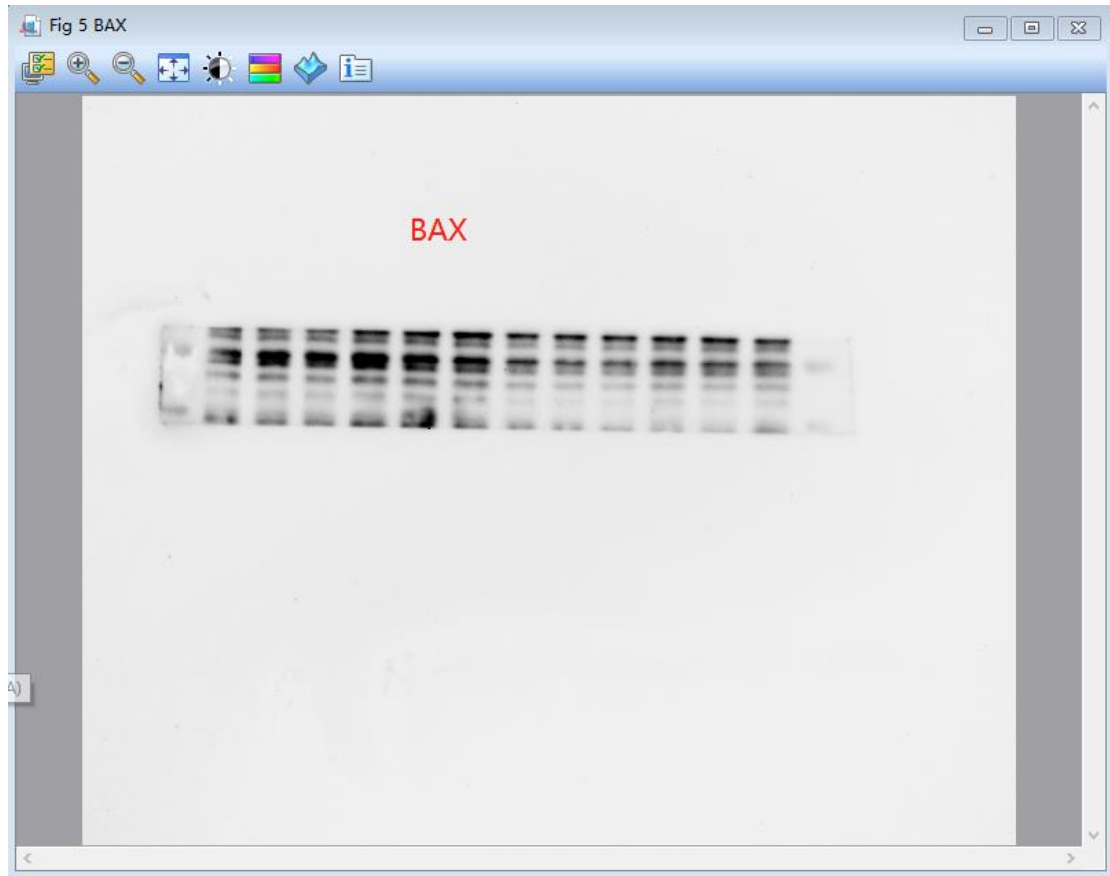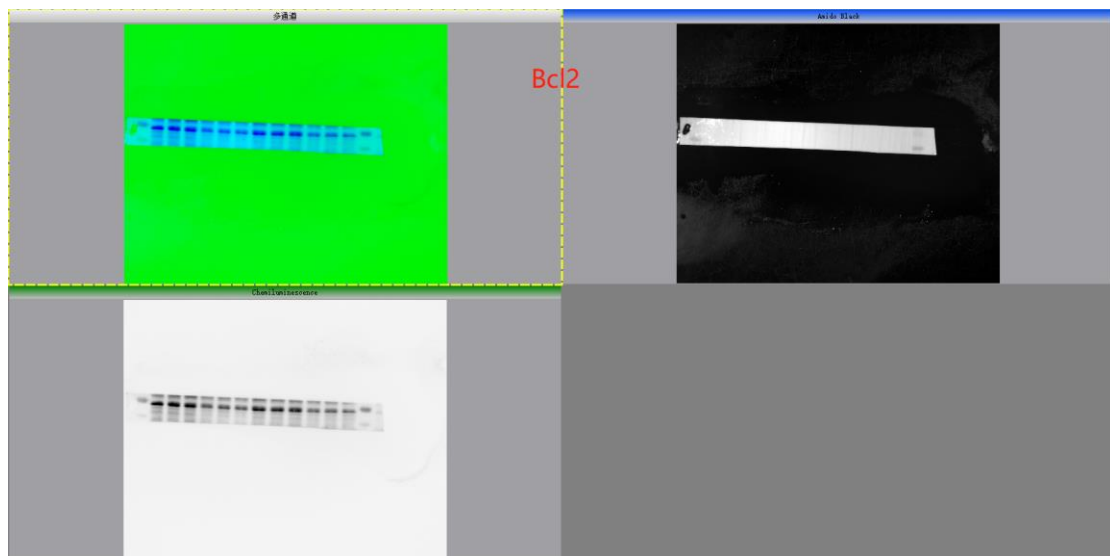

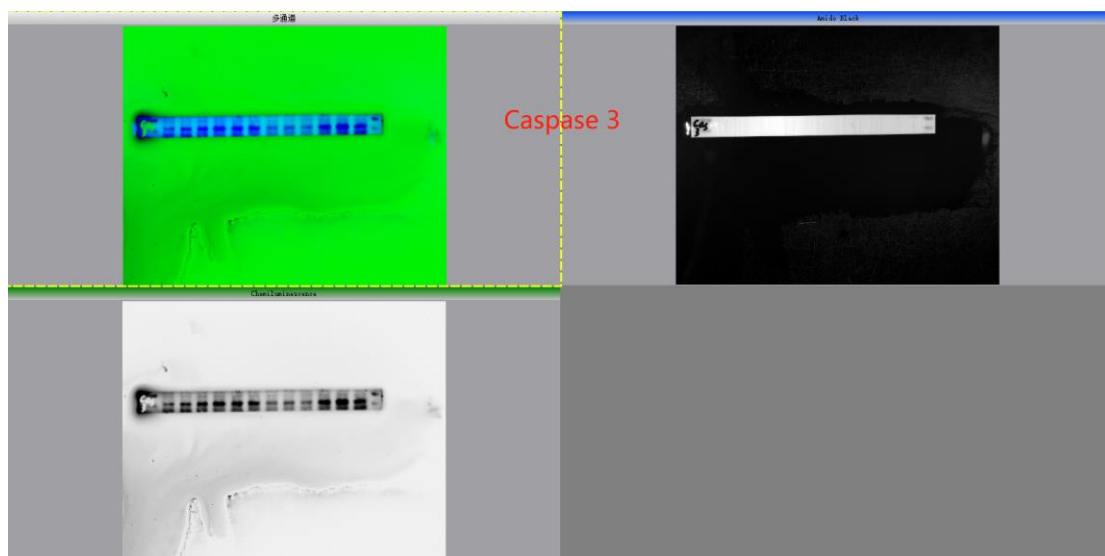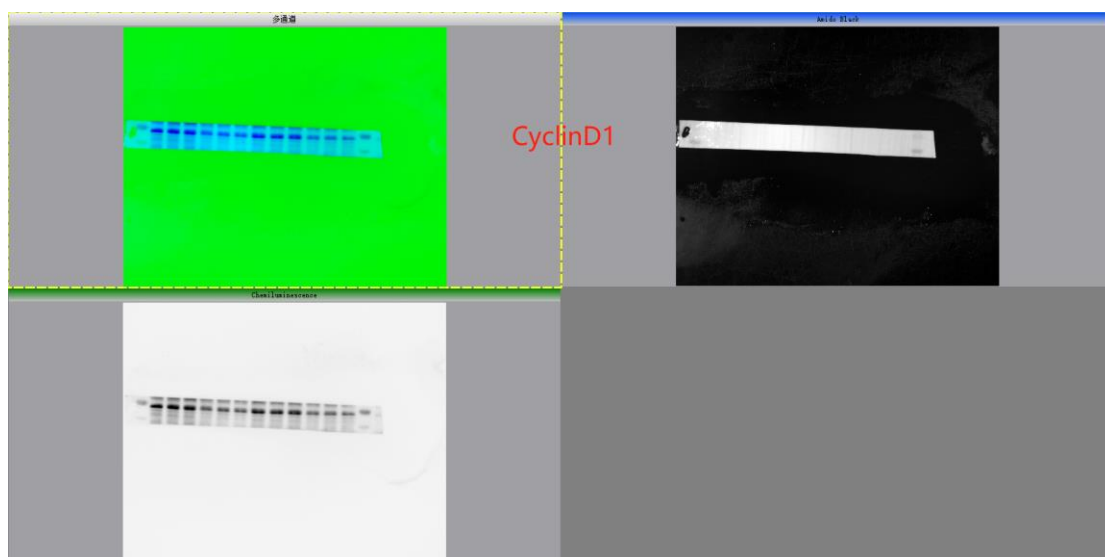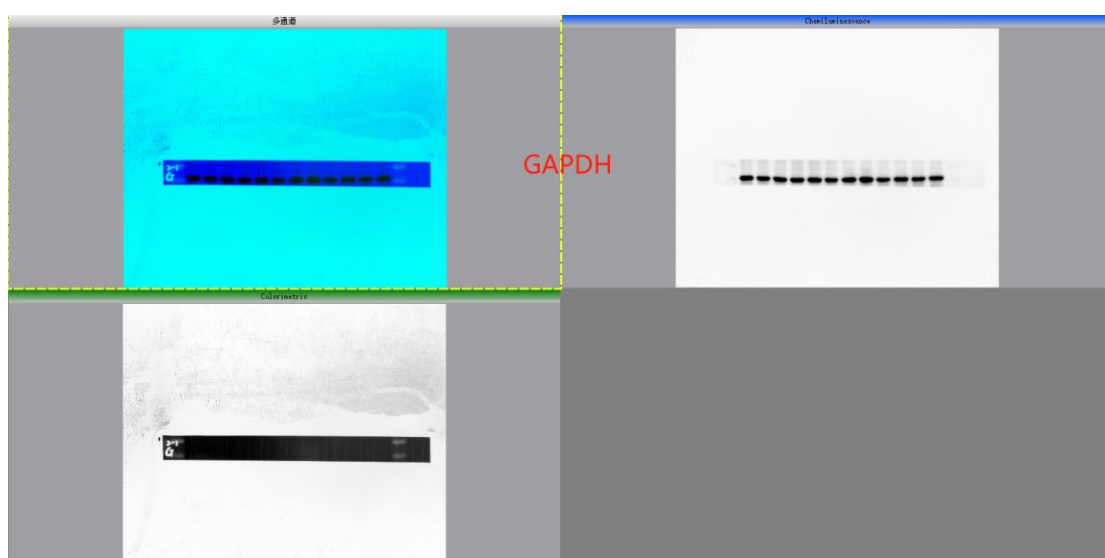

Supplement: Supplementary file 1 — Supplementary Material 1 [file 12935_2023_2997_MOESM1_ESM.pdf]

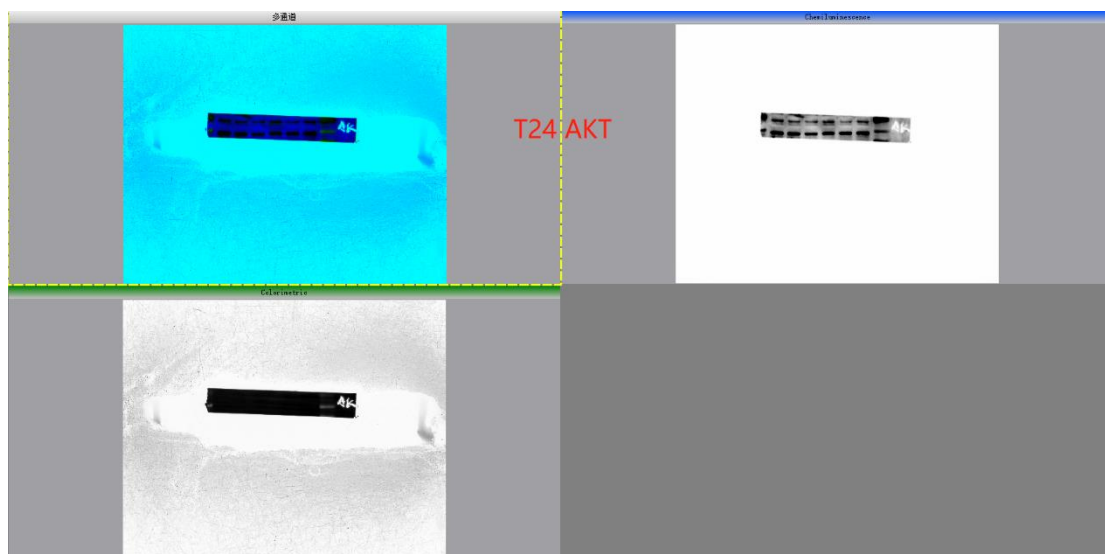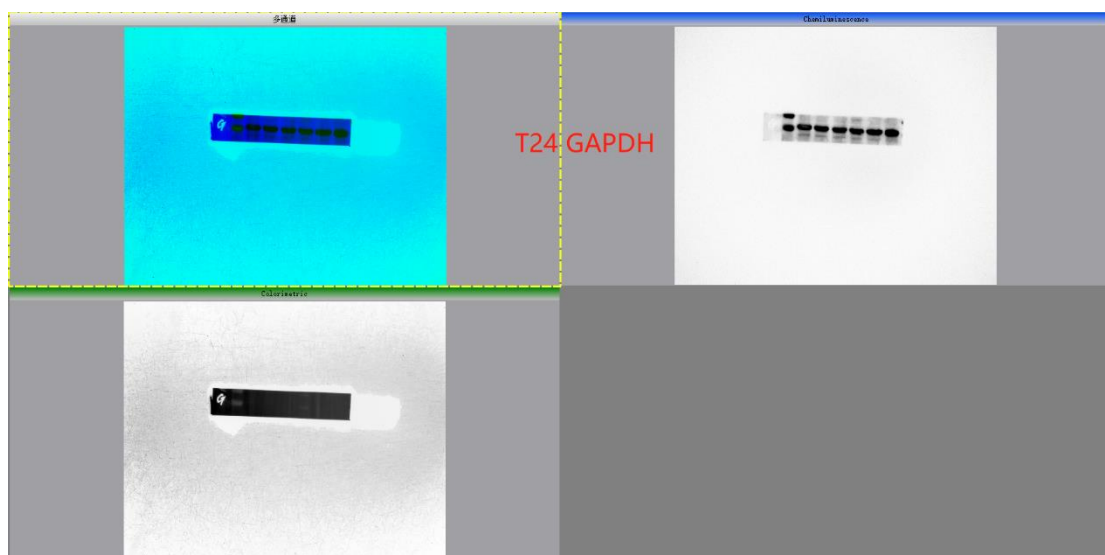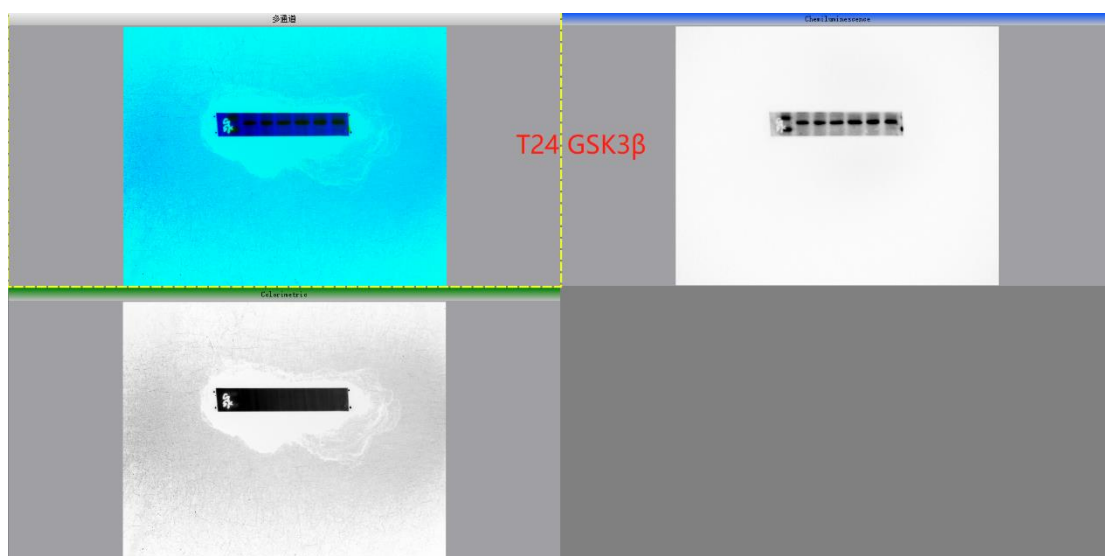

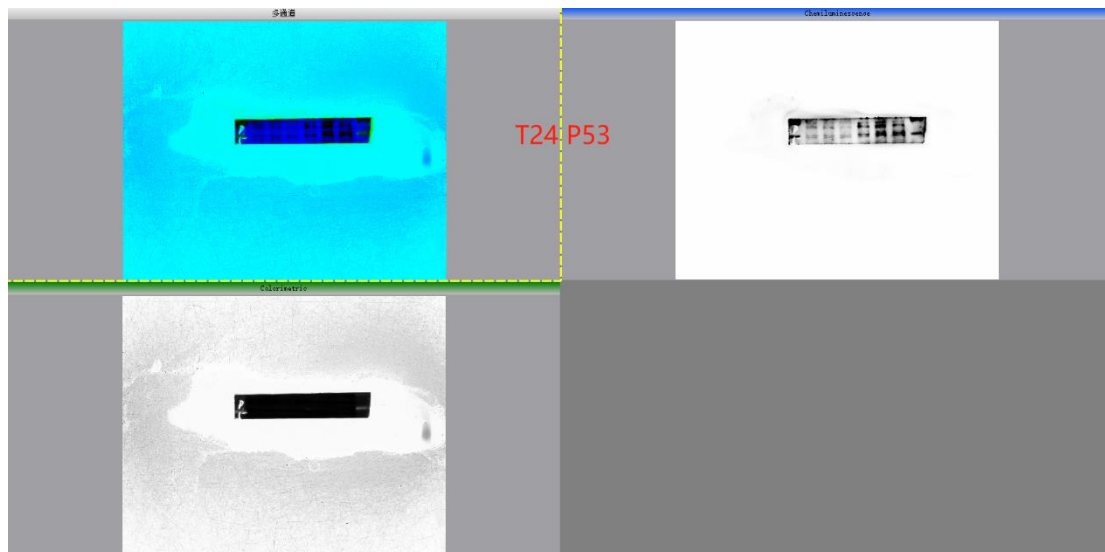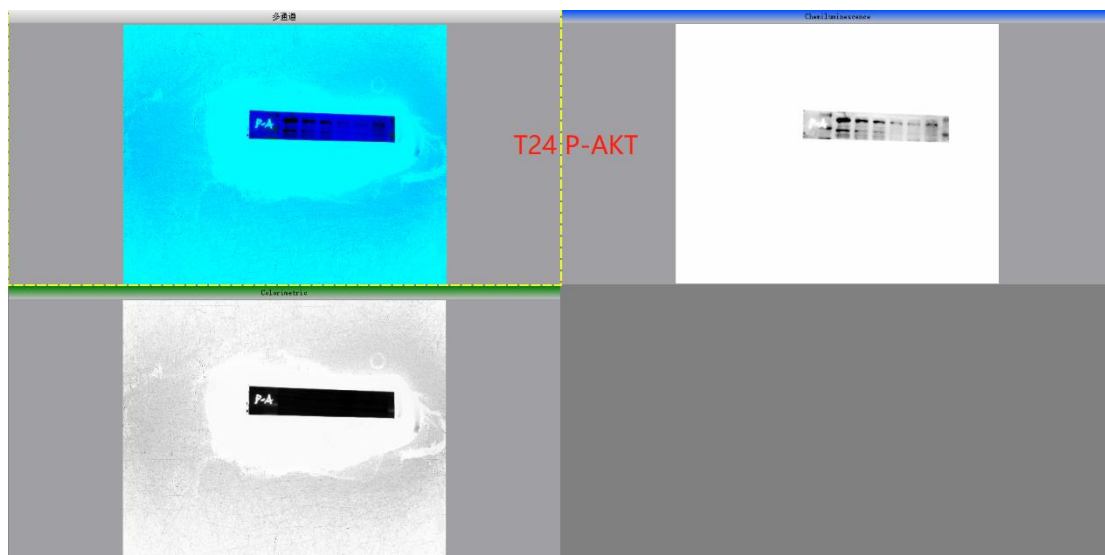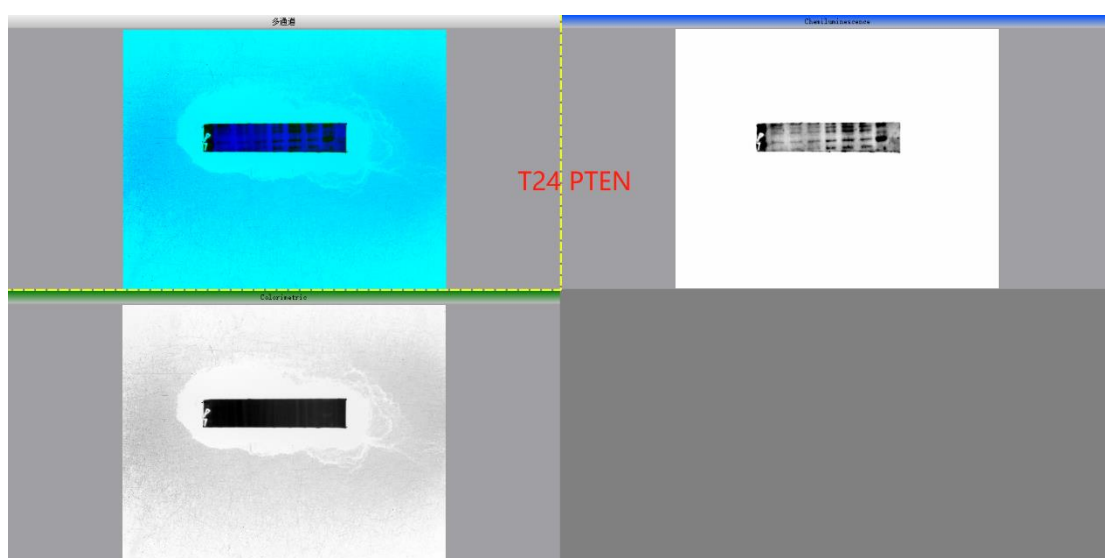

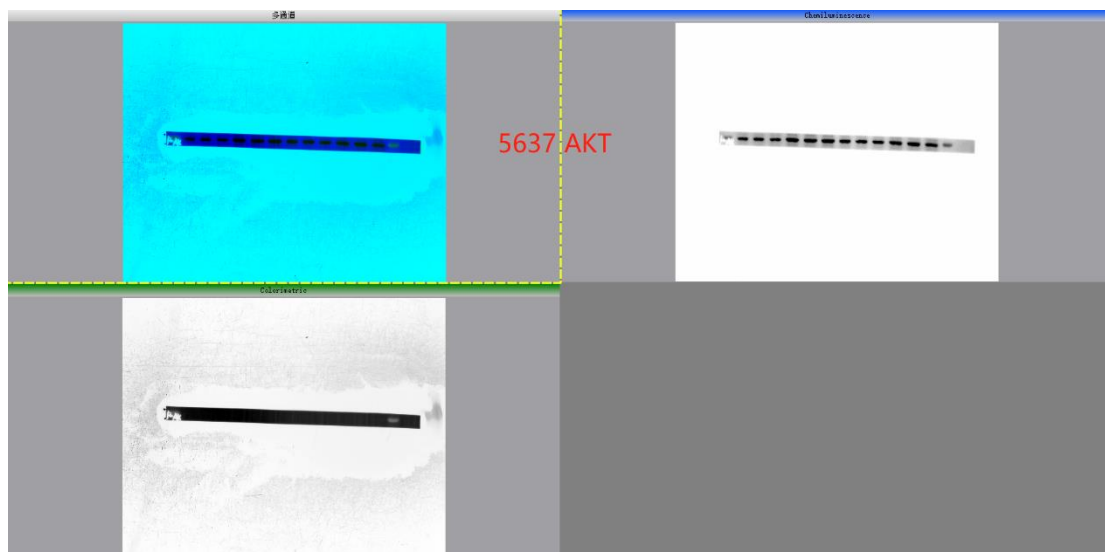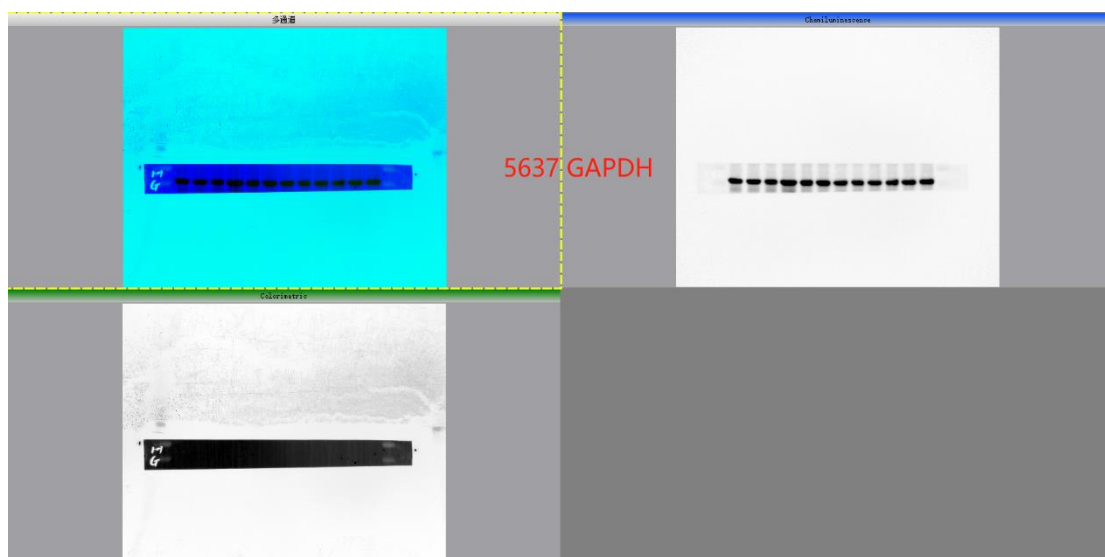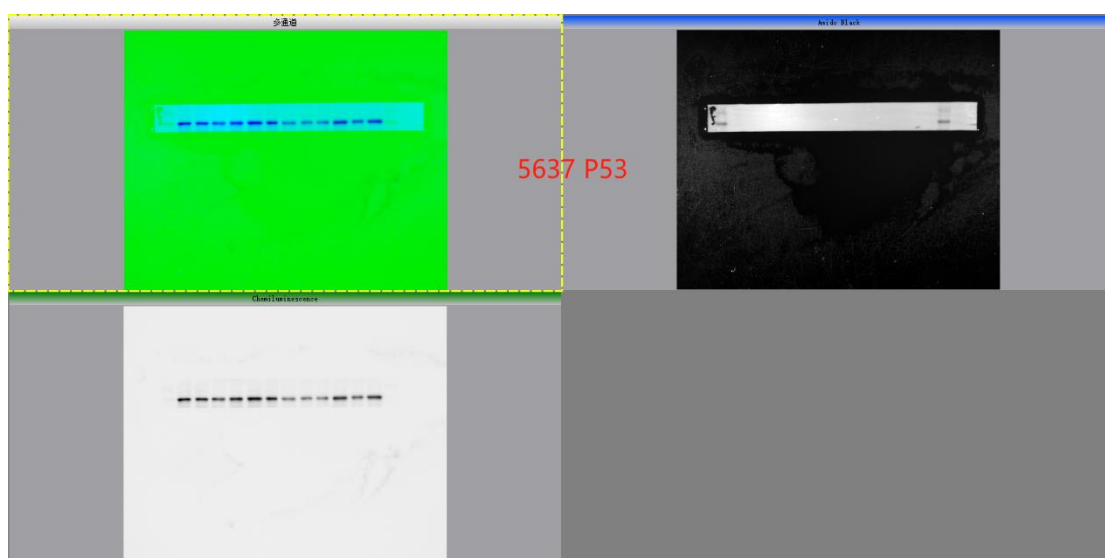

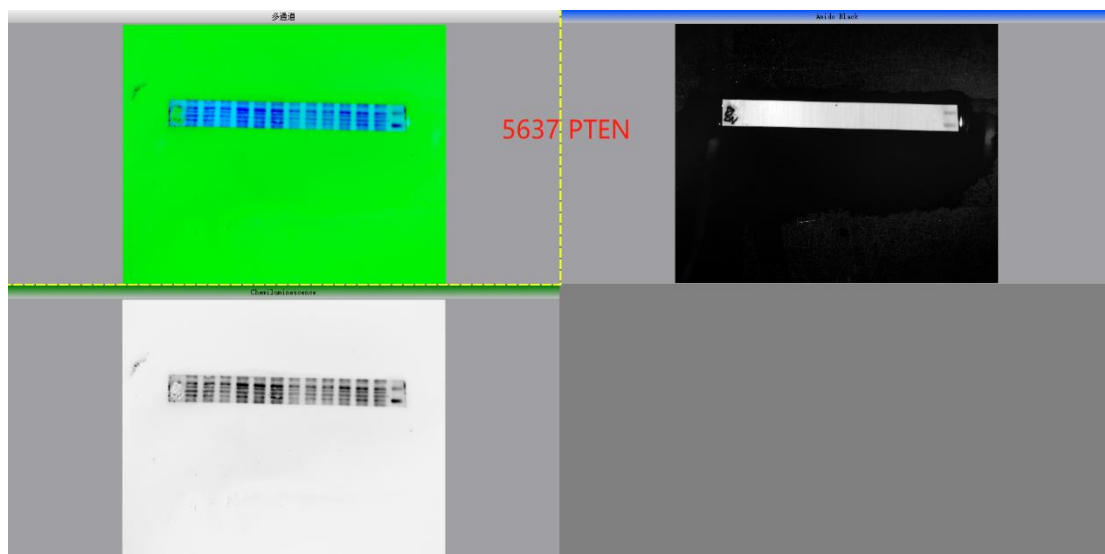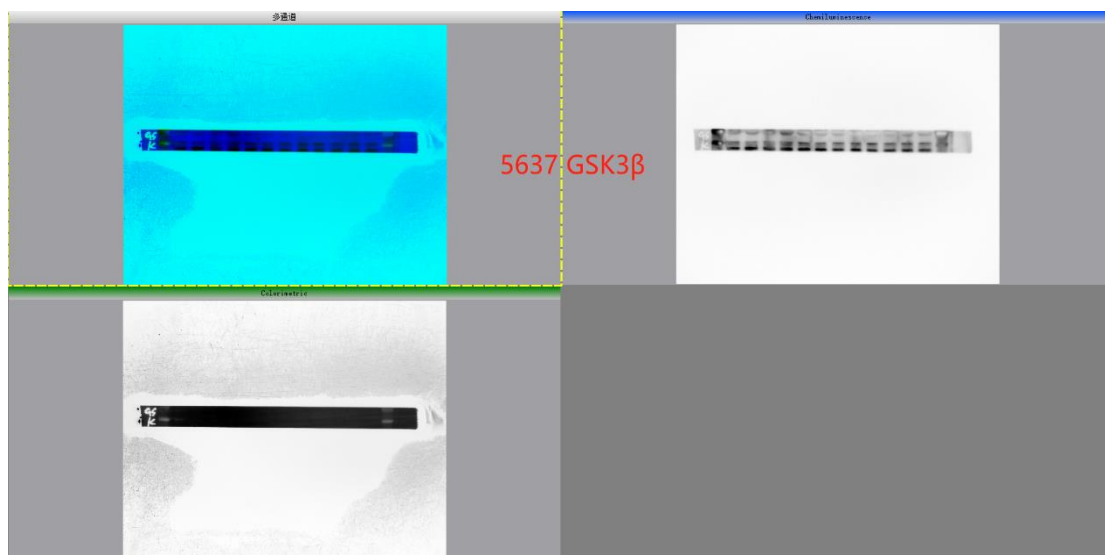

Supplement: Supplementary file 2 — Supplementary Material 2 [file 12935_2023_2997_MOESM2_ESM.pdf]

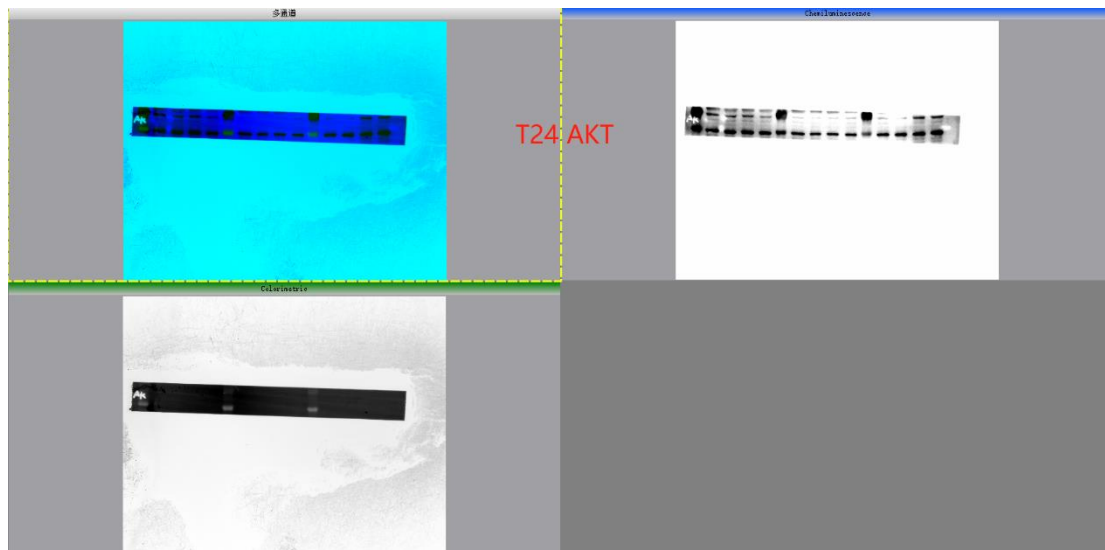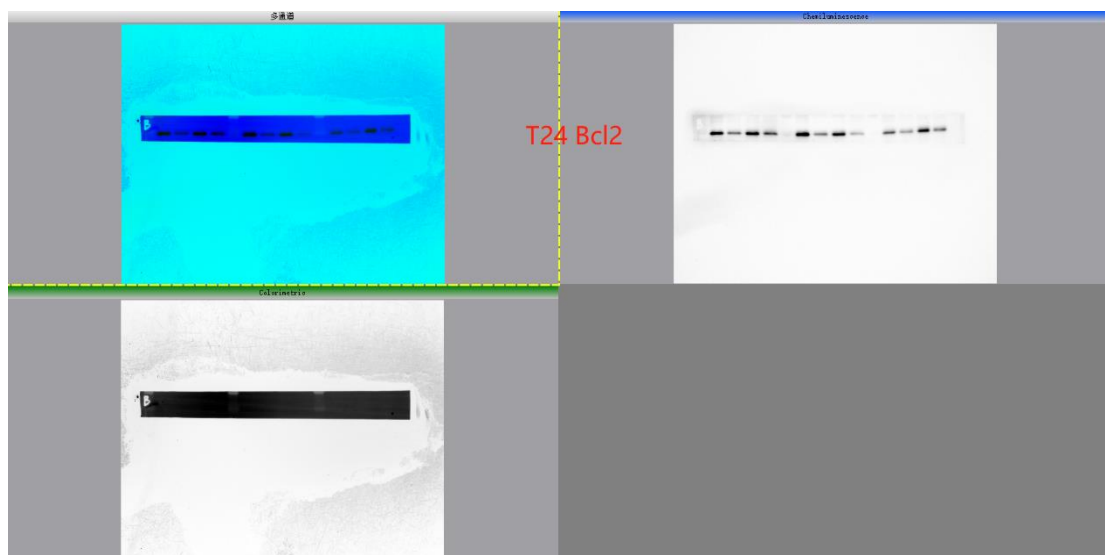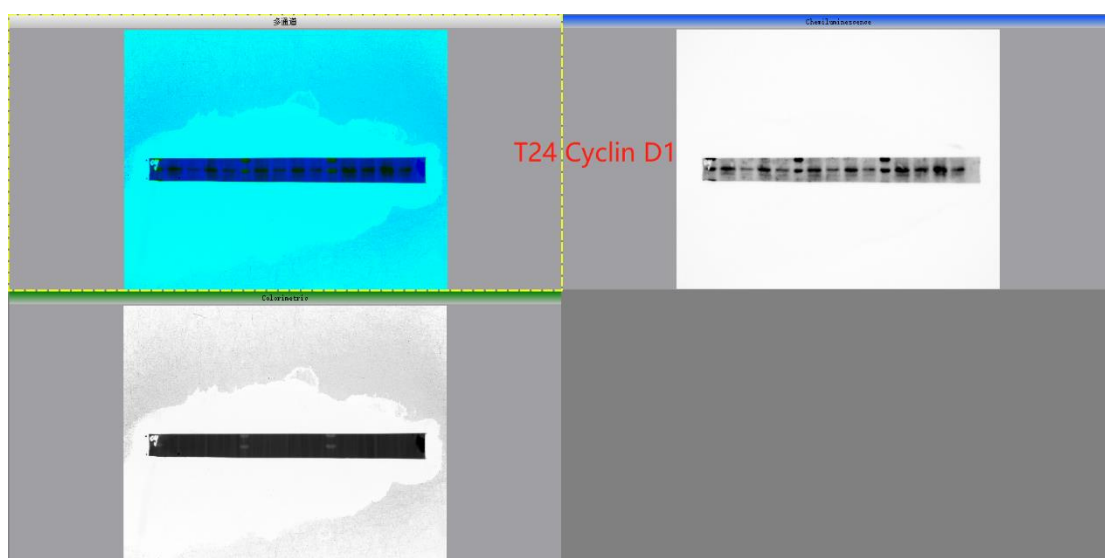

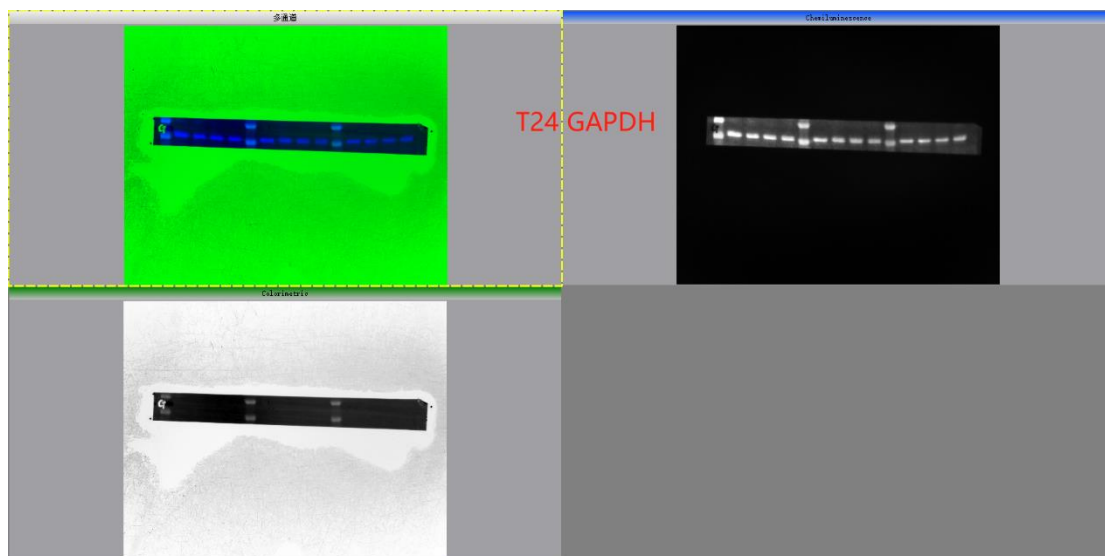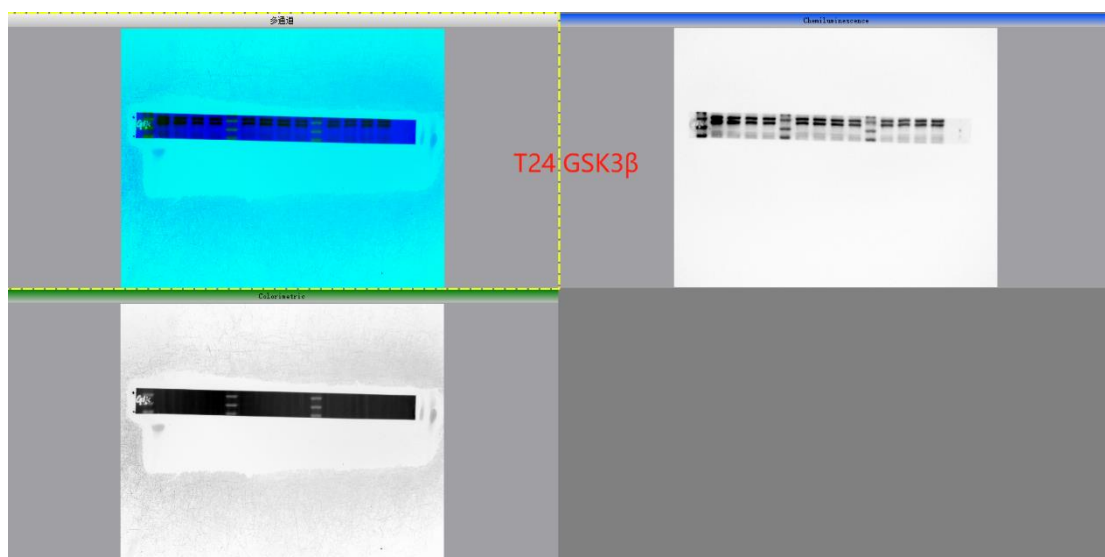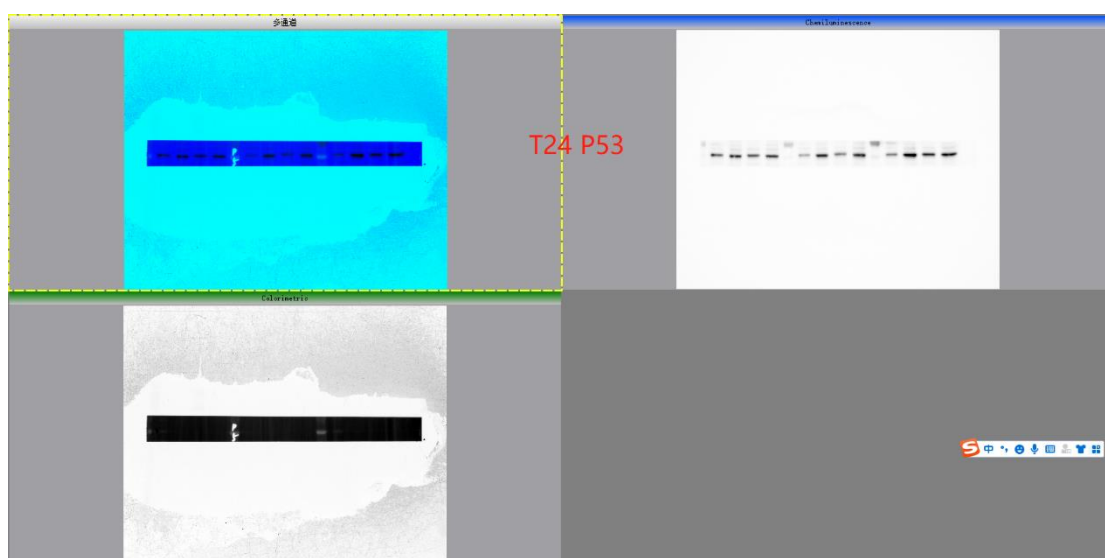

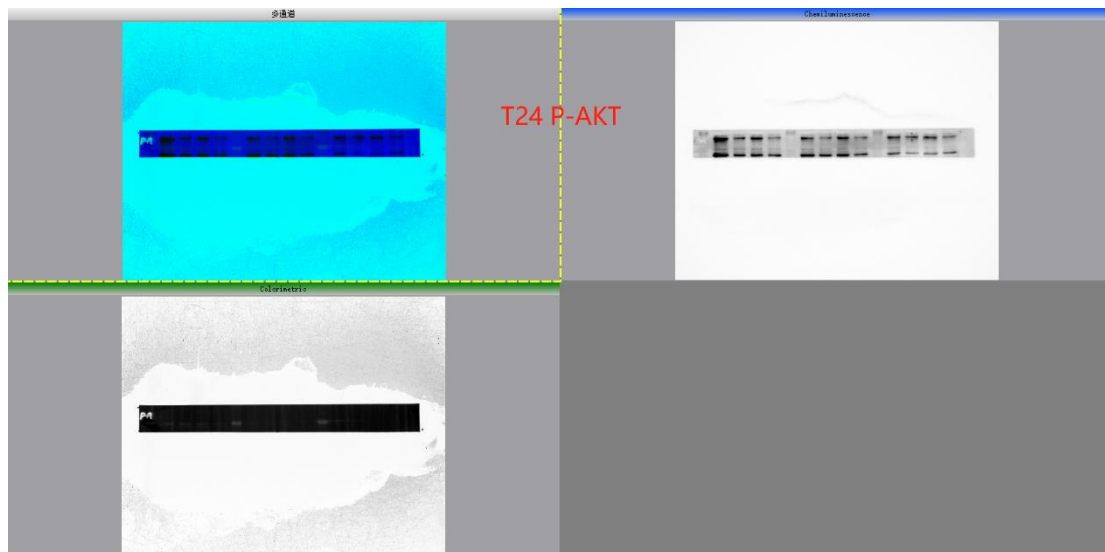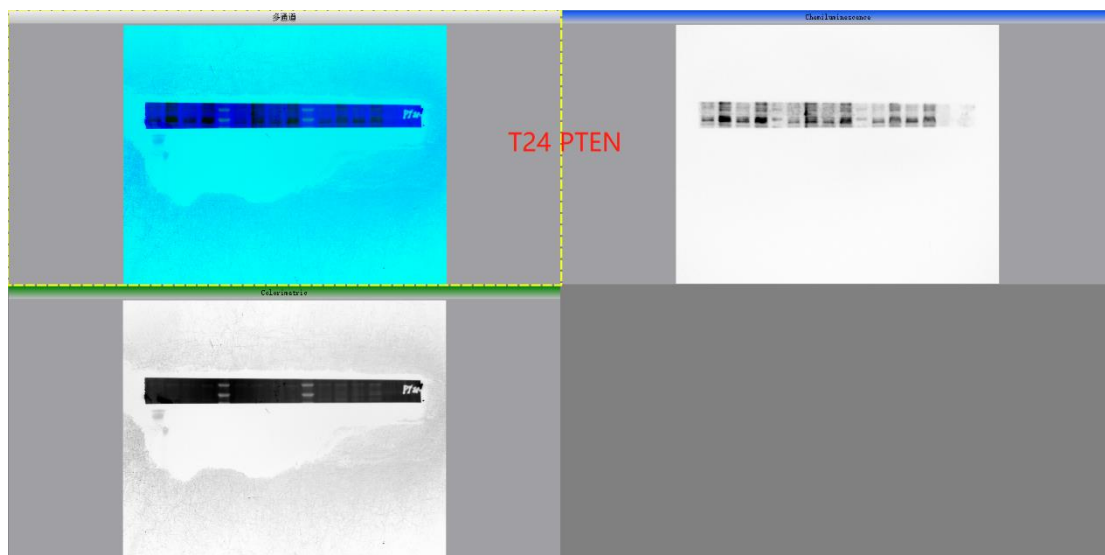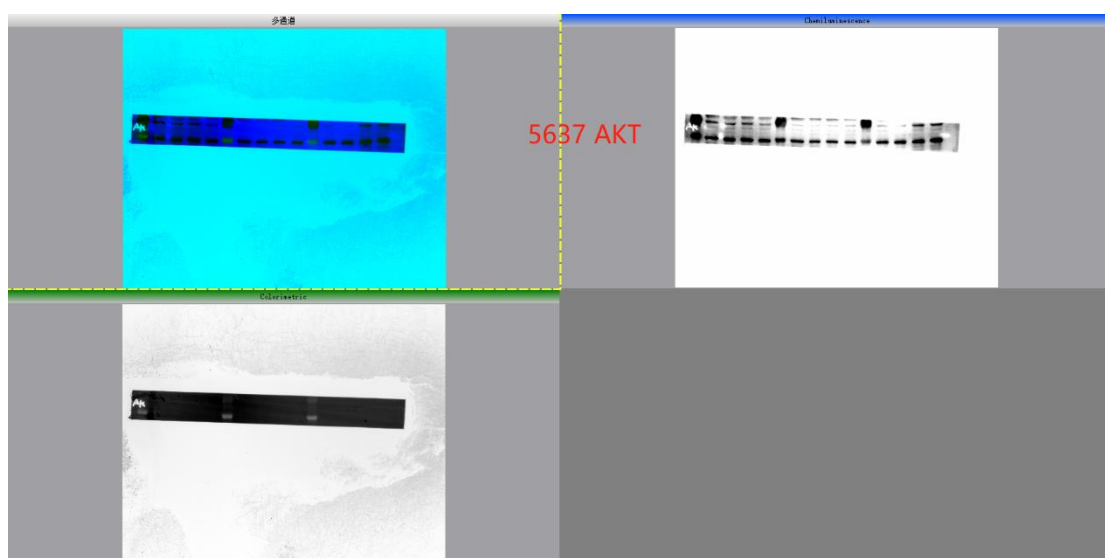

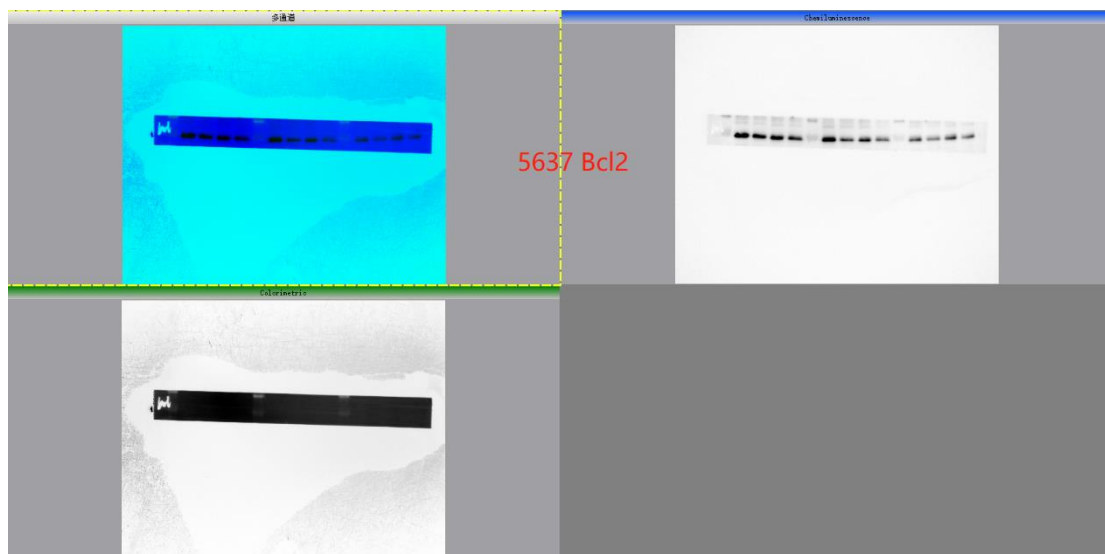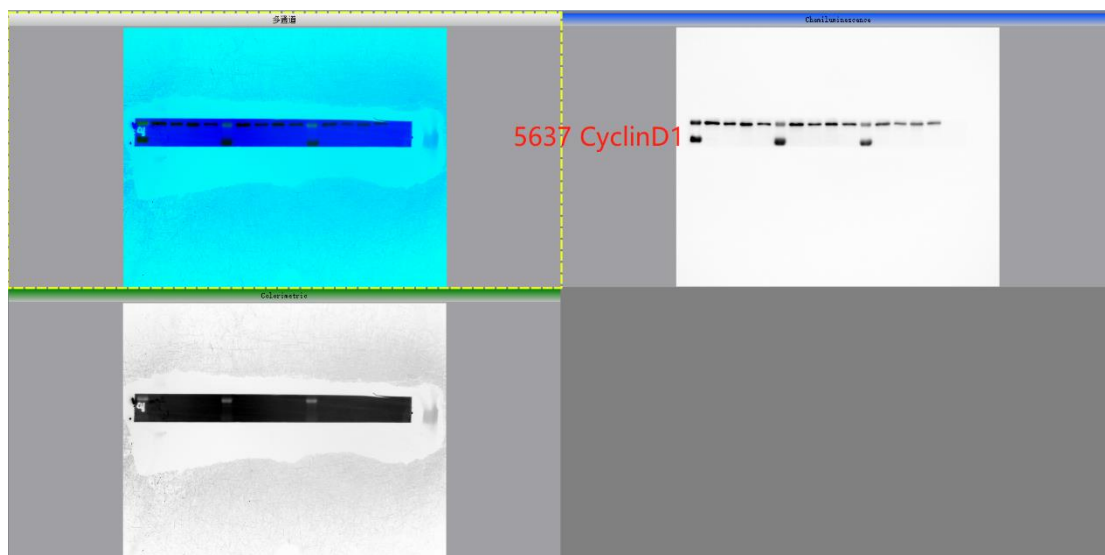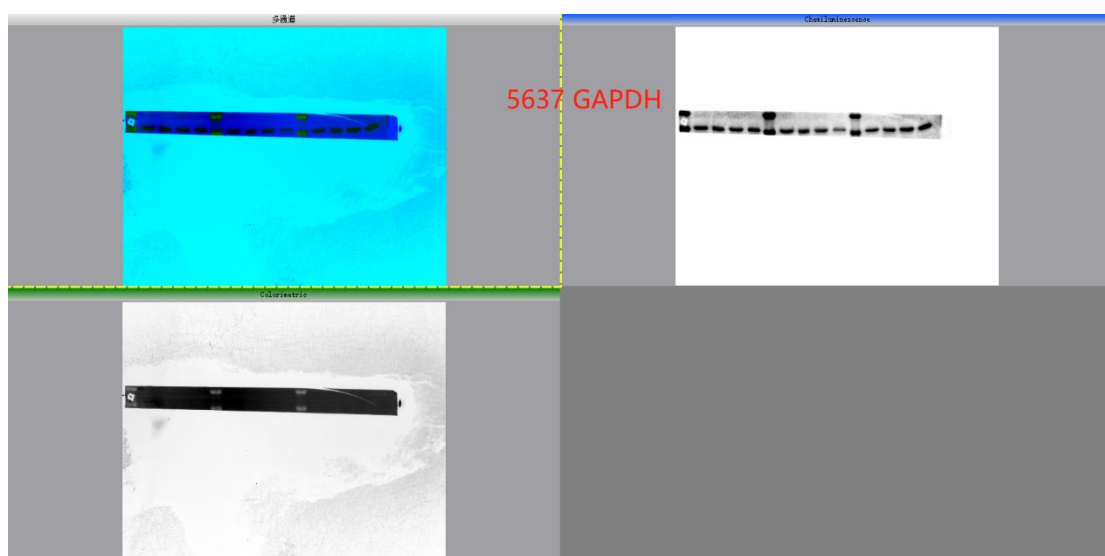

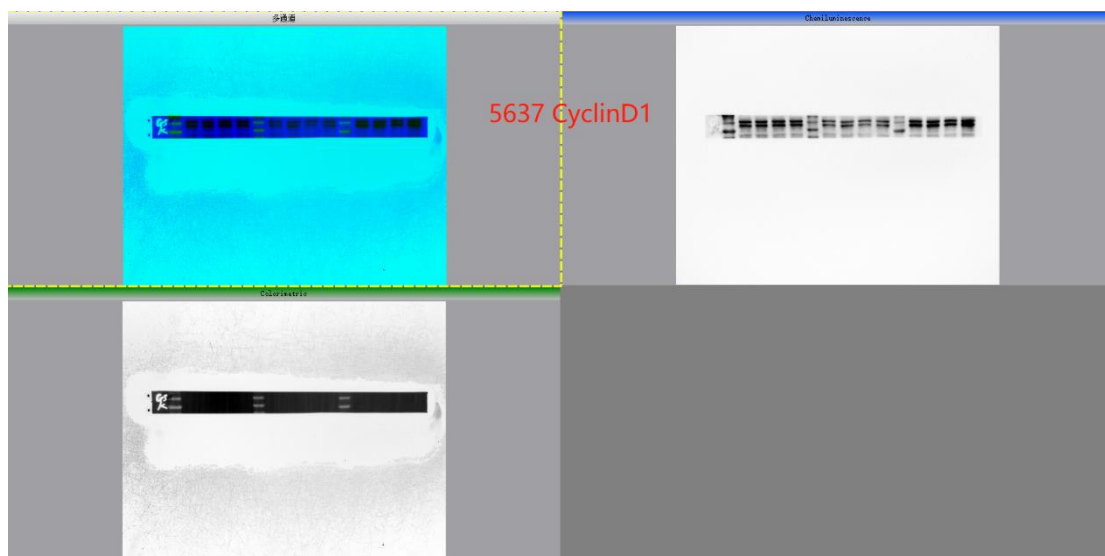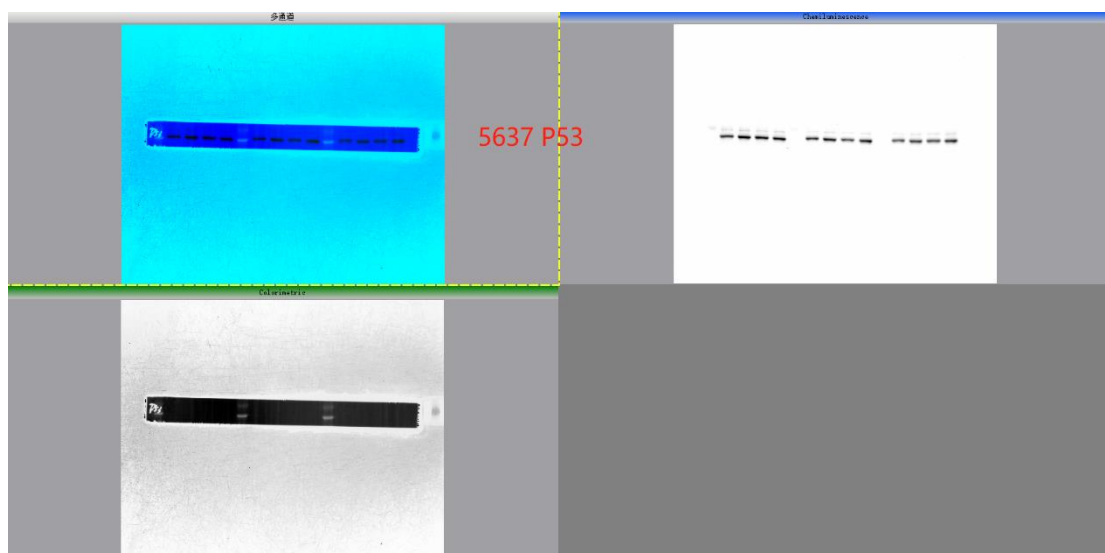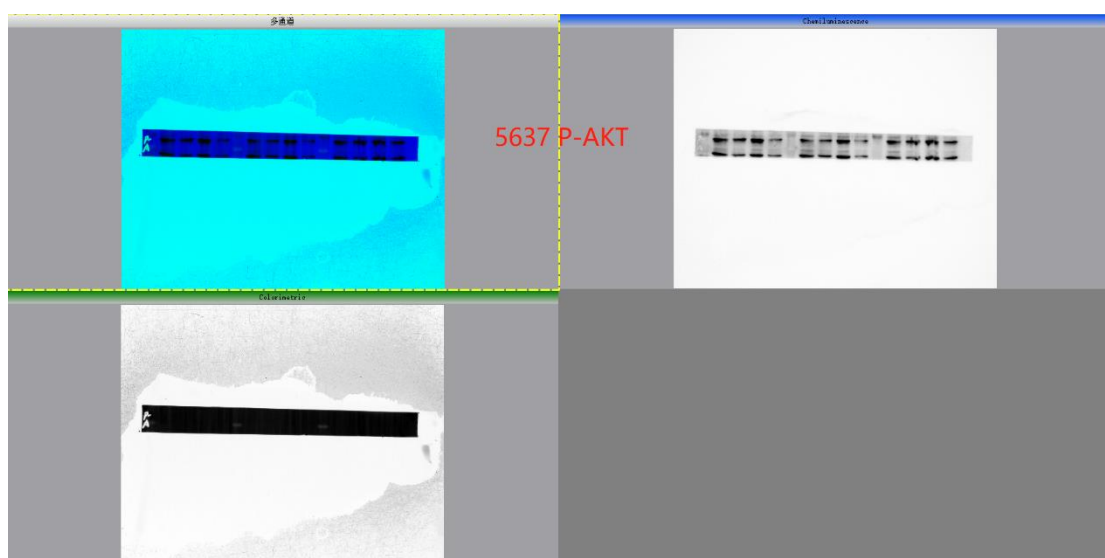

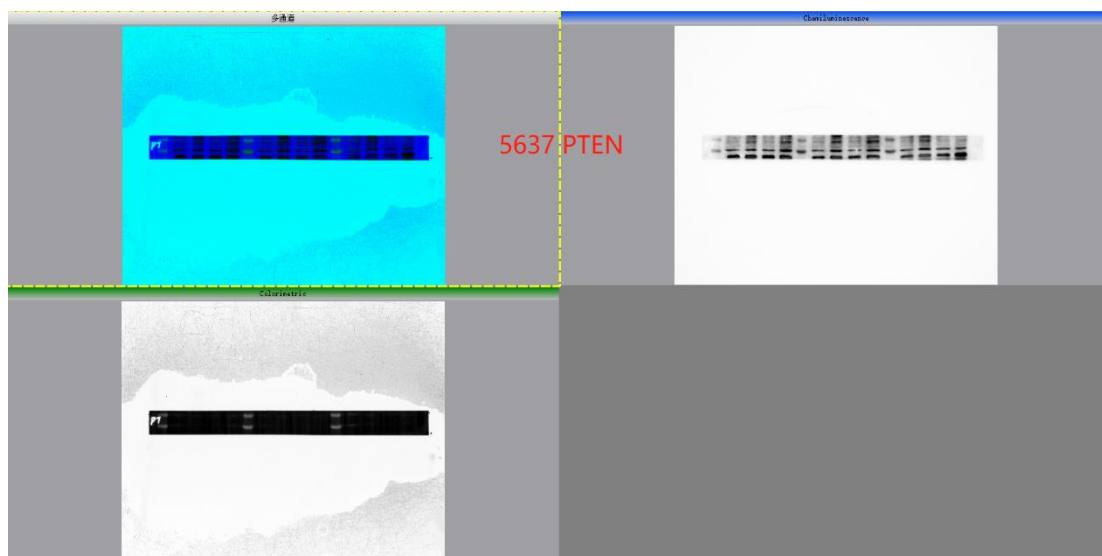

Supplement: Supplementary file 3 — Supplementary Material 3 [file 12935_2023_2997_MOESM3_ESM.pdf]

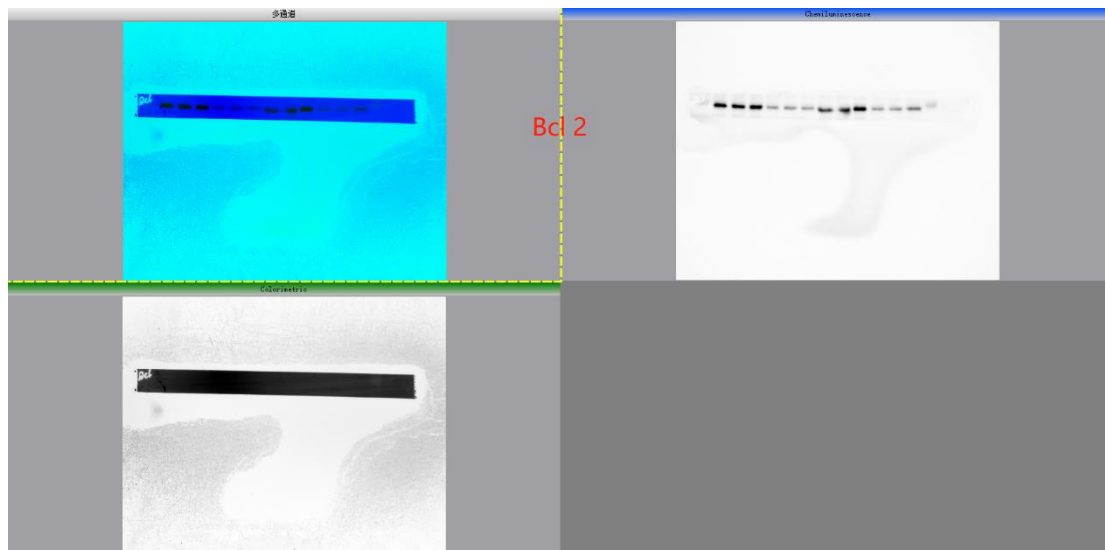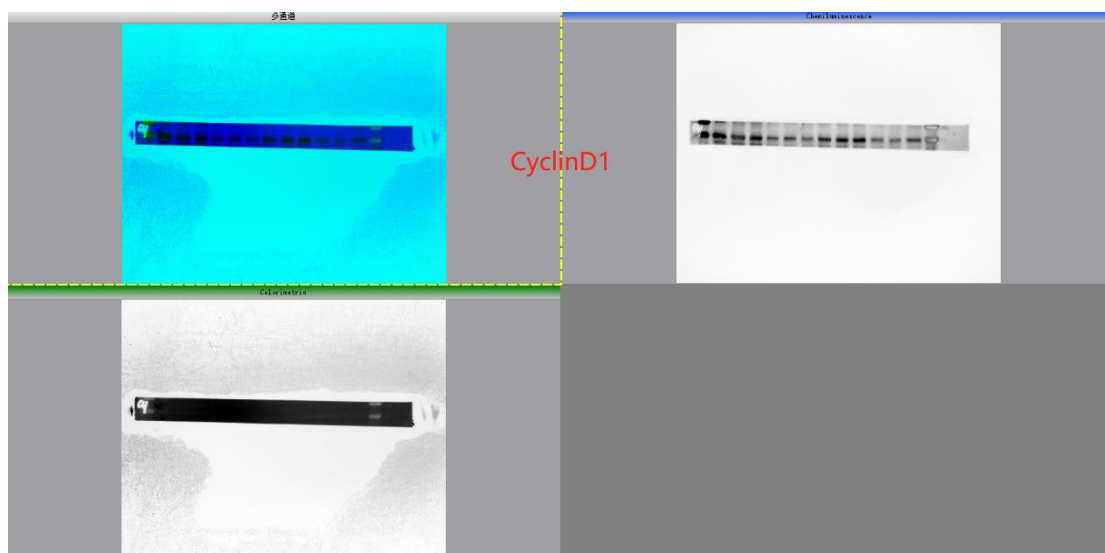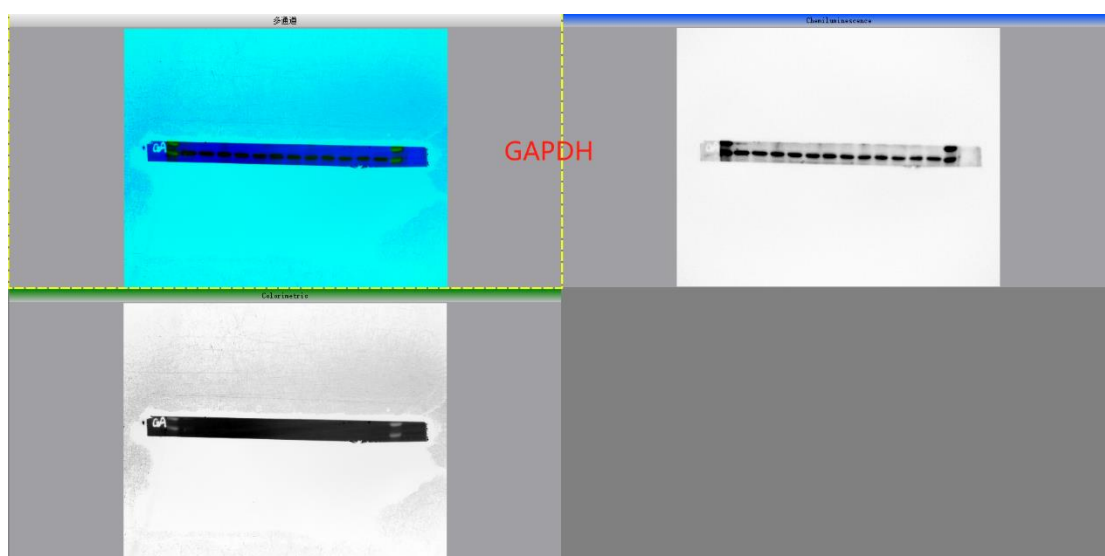

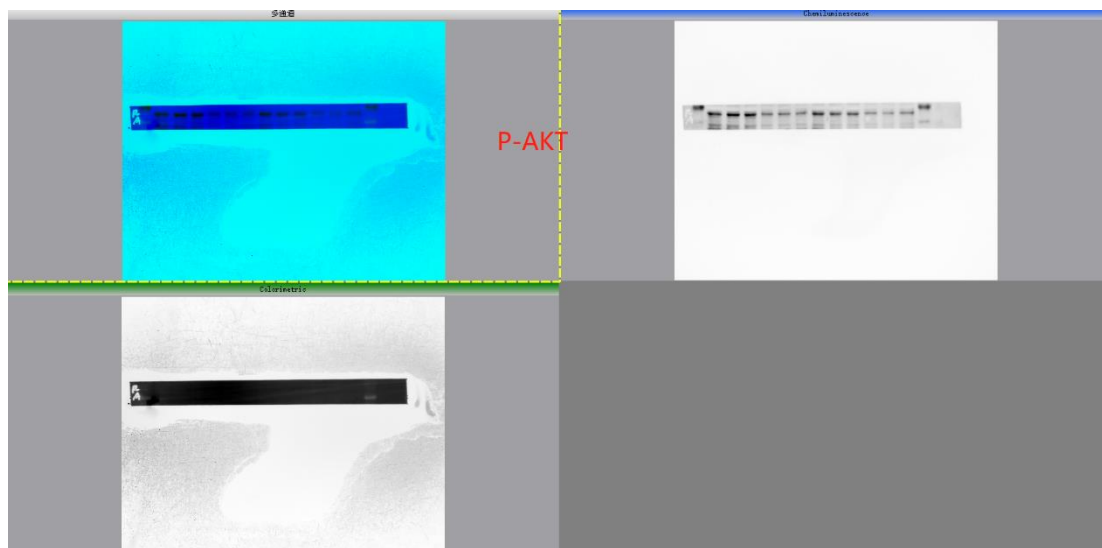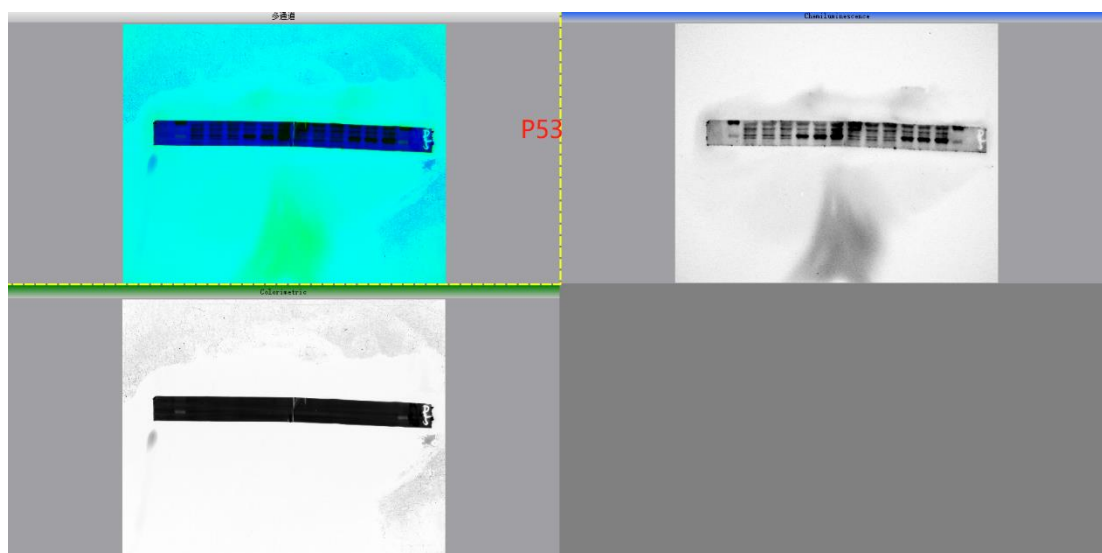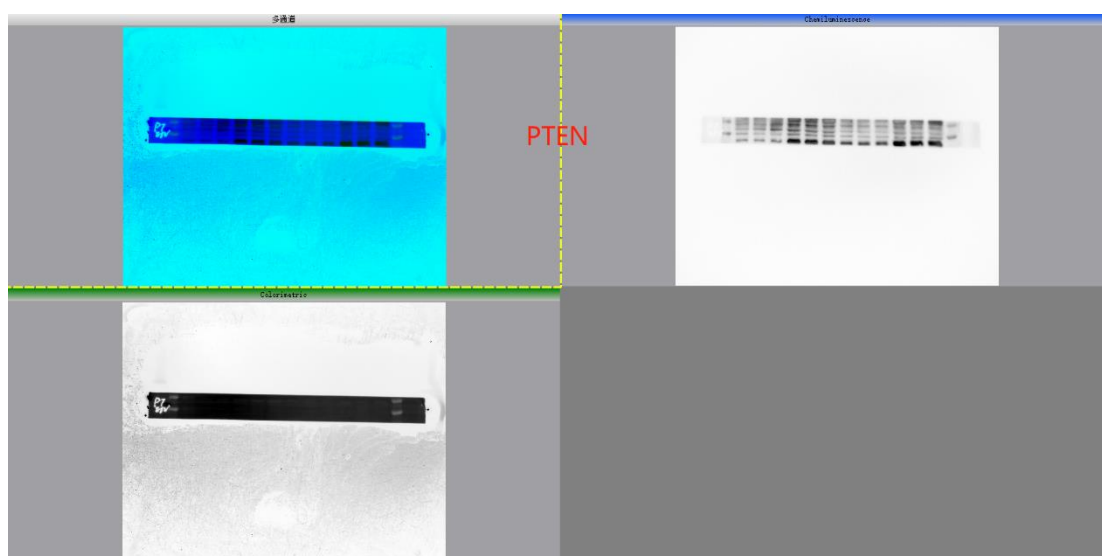

Supplement: Supplementary file 4 — Supplementary Material 4 [file 12935_2023_2997_MOESM4_ESM.pdf]
