# Supplementary material for: SQLE Knockdown inhibits bladder cancer progression by regulating the PTEN/AKT/GSK3β signaling pathway through P53
Source: Cancer Cell Int. 2023 Sep 28;23:221. doi: 10.1186/s12935-023-02997-5 (PMC10540347; doi:10.1186/s12935-023-02997-5)
Supplement: Supplementary file 5 — Supplementary Material 5 [file 12935_2023_2997_MOESM5_ESM.docx]

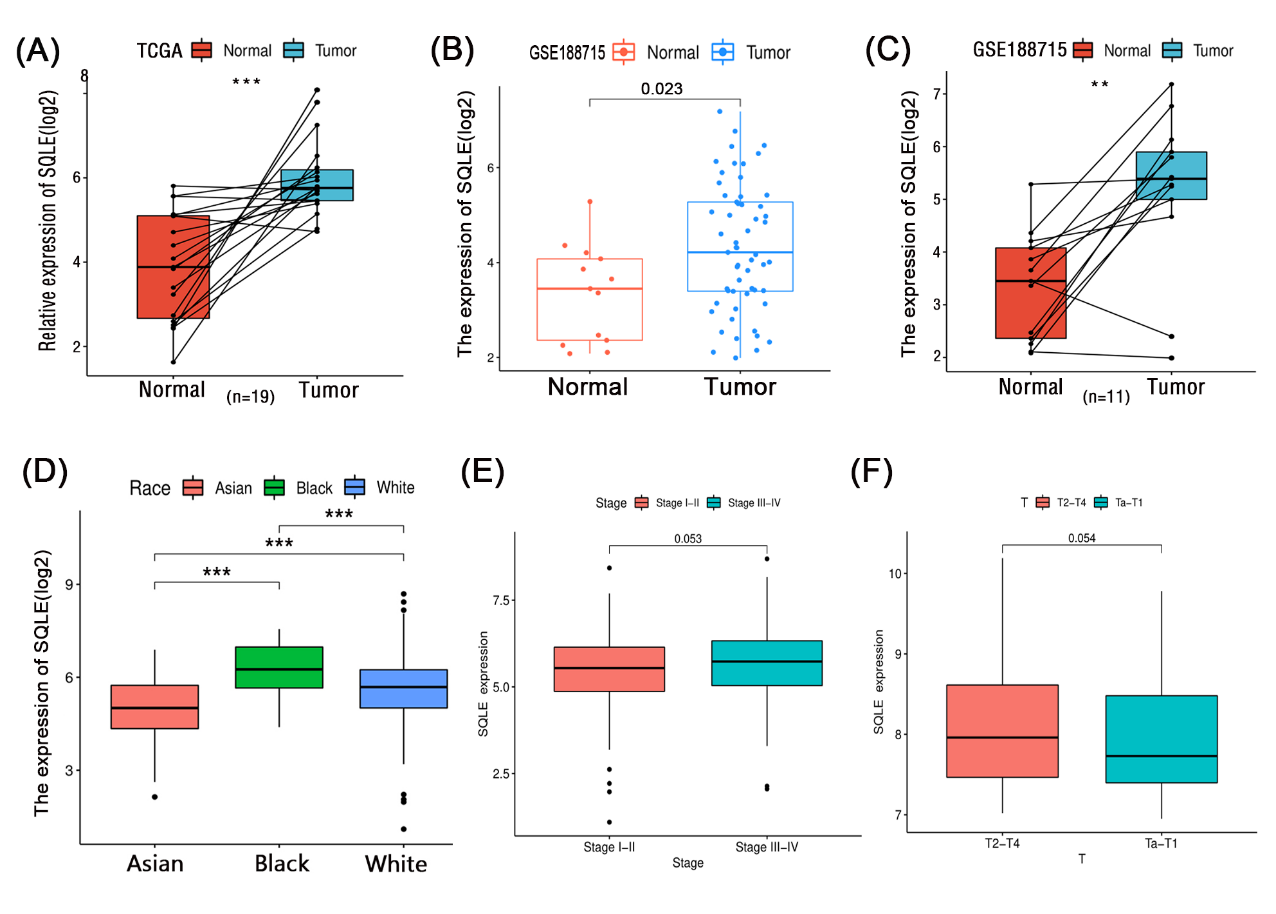


**Figure S1** | SQLE is highly expressed in tumor tissues and the expression of SQLE is associated with various clinical characters. (A) The mRNA expression level of SQLE in tumor tissue and its adjacent normal tissue in TCGA database; (B) The mRNA expression level of SQLE in all tumor tissues and adjacent normal tissues in TCGA database; (C) The mRNA expression level of SQLE in tumor tissue and its adjacent normal tissue in GSE188715 database; (D-F) The correlation between the expression of SQLE and some clinicopathological features of BCa. (*P<0.05, **P<0.01, ***P<0.001).


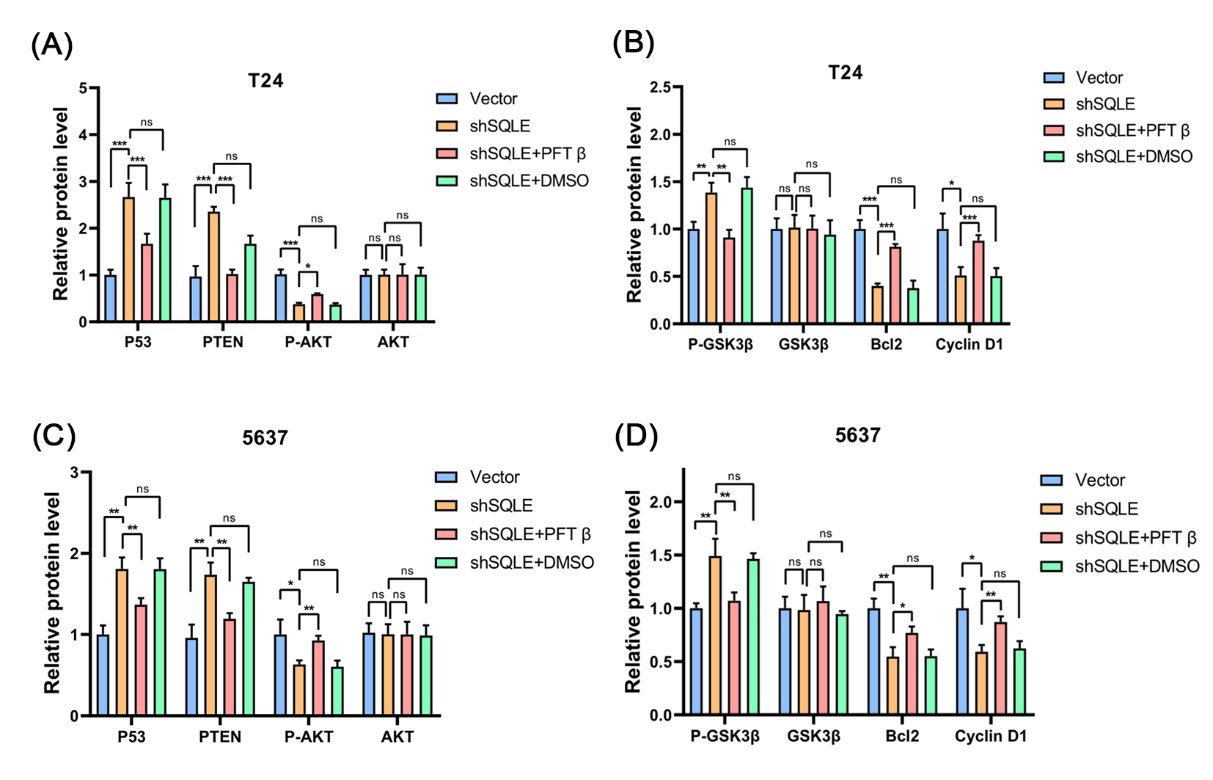


**Figure S2** | Protein expression levels of P53, PTEN, AKT, P-AKT, GSK3β, P-GSK3β, Bcl2, and Cyclin D1 in T24 and 5637 cells are shown in (A), (B), (C) and (D). Data represent the mean ± SD of three independent experiments. (*P<0.05, **P<0.01, ***P<0.001).
